# Urbanisation and asthma in low-income and middle-income countries: a systematic review of the urban–rural differences in asthma prevalence

**DOI:** 10.1136/thoraxjnl-2018-211793

**Published:** 2019-07-05

**Authors:** Alejandro Rodriguez, Elizabeth Brickley, Laura Rodrigues, Rebecca Alice Normansell, Mauricio Barreto, Philip J Cooper

**Affiliations:** 1 Faculty of Epidemiology and Population Health, London School of Hygiene and Tropical Medicine, London, UK; 2 Facultad de Ciencias Médicas de la Salud y la Vida, Universidad Internacional del Ecuador, Quito, Ecuador; 3 Fundación Ecuatoriana para la Investigación en Salud, Quito, Ecuador; 4 Population Health Research Institute, London, UK; 5 Instituto de Saude Coletiva, Universidad Federal da Bahia, Salvador, Brazil; 6 Centrode de Integração de Dados e Conhecimentos para Saúde (CIDACS), FIOCRUZ, Salvador, Brazil; 7 Institute of Infection and Immunity, St George’s University of London, London, UK

**Keywords:** urbanisation, asthma, urban and rural areas, LMICs

## Abstract

**Background:**

Urbanisation has been associated with temporal and geographical differences in asthma prevalence in low-income and middle-income countries (LMICs). However, little is known of the mechanisms by which urbanisation and asthma are associated, perhaps explained by the methodological approaches used to assess the urbanisation-asthma relationship.

**Objective:**

This review evaluated how epidemiological studies have assessed the relationship between asthma and urbanisation in LMICs, and explored urban/rural differences in asthma prevalence.

**Methods:**

Asthma studies comparing urban/rural areas, comparing cities and examining intraurban variation were assessed for eligibility. Included publications were evaluated for methodological quality and pooled OR were calculated to indicate the risk of asthma in urban over rural areas.

**Results:**

Seventy articles were included in our analysis. Sixty-three compared asthma prevalence between urban and rural areas, five compared asthma prevalence between cities and two examined intraurban variation in asthma prevalence. Urban residence was associated with a higher prevalence of asthma, regardless of asthma definition: current-wheeze OR:1.46 (95% CI:1.22 to 1.74), doctor diagnosis OR:1.89 (95% CI:1.47 to 2.41), wheeze-ever OR:1.44 (95% CI:1.15 to 1.81), self-reported asthma OR:1.77 (95% CI:1.33 to 2.35), asthma questionnaire OR:1.52 (95% CI:1.06 to 2.16) and exercise challenge OR:1.96 (95% CI:1.32 to 2.91).

**Conclusions:**

Most evidence for the relationship between urbanisation and asthma in LMICs comes from studies comparing urban and rural areas. These studies tend to show a greater prevalence of asthma in urban compared to rural populations. However, these studies have been unable to identify which specific characteristics of the urbanisation process may be responsible. An approach to understand how different dimensions of urbanisation, using contextual household and individual indicators, is needed for a better understanding of how urbanisation affects asthma.

**PROSPERO registration number:**

CRD42017064470.

**Key messages**

**What is the key question?**

- The effects of urbanisation on asthma prevalence in low-income and middle-income countries (LMICs).

**What is the bottom line?**

- Asthma prevalence is greater in urban than rural populations in LMICs, but the mechanisms by which urbanisation affects asthma are not clear, explained probably by the methods used to measure urbanisation.

**Why read on?**

- Our systematic review provides evidence that urban residence and urbanisation are important determinants of asthma prevalence although such studies to date have used inadequate methodological approaches to identify the causal factors involved.

## Introduction

The prevalence of asthma and related allergic disorders (RAD) has been increasing over the past four decades.^1^ However, recent evidence indicates that the prevalence may have reached a plateau in high-income countries (HICs) with a high prevalence, but continues to increase in lower prevalence LMICs, particularly among urban populations.^2 3^ The factors that underlie such temporal and geographical trends in asthma prevalence are poorly understood, but are likely to reflect a complex interplay of biologic, environmental and social factors.^4^

It has been hypothesised that the urbanisation process could be in part responsible for the temporal and geographical variations of asthma prevalence in both HICs and LMICs.^5–7^ This hypothesis has received support mainly by three observations. First, studies on wheezing or asthma in different regions of the world have regularly shown a lower asthma prevalence in rural settlements compared with cities.^5 8 9^ Second, the low asthma prevalence in rural areas has been explained by possible protection provided by traditional rural exposures such as farming.^6 7^ However, recent studies have shown that allergic disorders could be increasing in rural areas, reducing the urban–rural gap in asthma prevalence.^10–12^ Third, exposures relating to environmental and lifestyle changes that originate from the urbanisation process have been identified as risk factors for asthma including changes in diet, sedentarism, reductions in childhood infections, smaller families, use of antibiotics, environmental pollution and migration.^6 13^

Epidemiological studies have provided invaluable information about the relationship between urbanisation and asthma through use of diverse methods and indicators of urbanisation. However, studies evaluating the effects of urbanisation on asthma are complex and face several conceptual and methodological limitations. First, there is no standard definition of urbanisation. Urbanisation is a highly complex process that affects all levels of human activity and no single definition can fully describe the multidimensional nature of this process.^14^ Second, there is no universally accepted definition of what constitutes an urban area. Different countries use different definitions for urban areas mainly based on demographic, political or economic characteristics of their populations.^15^ Third, there is no agreed definition of asthma for research purposes, so different studies use different definitions such as doctor diagnosis, presence of clinical symptoms and bronchial hyper-responsiveness.^16 17^

In LMICs, the specific features and mechanisms by which urbanisation affects asthma are not clear. Part of this problem may lie in the methods used by asthma studies to assess the effects of urbanisation on asthma. The aim of this systematic review is to provide a general overview of how epidemiological studies have assessed the relationship between asthma prevalence and urbanisation in LMICs.

In this review, we addressed the following research objectives:

1. To examine the methods used to evaluate the effects of urbanisation on asthma.
2. To examine rural ⁄urban differences in asthma prevalence.

## Methods

We performed a systematic review of the scientific literature to identify studies that have assessed the relationship between asthma and urbanisation in LMICs following Preferred Reporting Items for Systematic Reviews and Meta-Analyses guidelines.^18^

### Inclusion criteria

#### Population and context

Subjects of all ages living in urban or rural areas of LMICs. We excluded populations living in HICs. LMICs were defined using the list of countries of the World Bank (https://datahelpdesk.worldbank.org) based on the year in which each study was conducted.

#### Study designs

Cross-sectional, case–control, cohort and ecological studies. We excluded intervention, experimental and genetic studies. Studies that lacked essential data for calculating ORs were also excluded.

#### Exposure

Urban areas or urban environments defined by demographic, socioeconomic, administrative or other indicators associated to the urbanisation process. We excluded studies evaluating the effects of air pollution on asthma.

#### Outcomes

Prevalence of asthma measured by wheeze/asthma in the last 12 months, clinical symptoms, doctor’s diagnosis, questionnaire data and pulmonary function tests.

### Search strategy

A literature search was done in PubMed, ScienceDirect and Scielo databases in February 2017 (online supplementary figure 1). To include all available evidence, past reviews, letters to the editor and publications discussing the relationship between urbanisation and asthma were also evaluated. Further, no restrictions were imposed regarding sample size, age, sex and publication date. Articles in English, Spanish and Portuguese were included in the search. The search process concluded on July 2017.

10.1136/thoraxjnl-2018-211793.supp1 Supplementary data

### Paper selection and retrieval process

**Figure 1:**
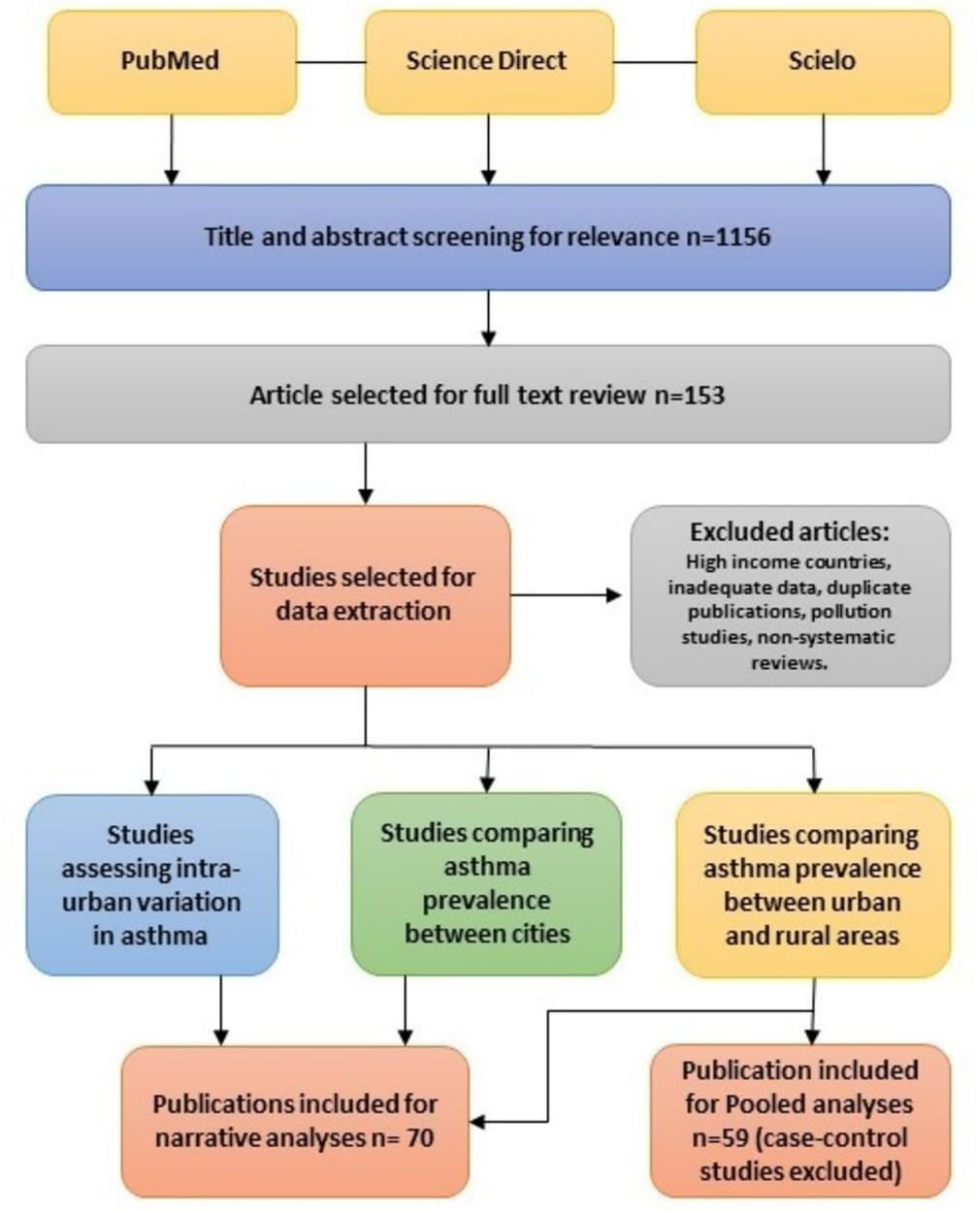
Flow chart of publication selection process.

Publications were grouped by three methods: (a) studies comparing the prevalence of asthma between rural and urban areas, (b) studies comparing the prevalence of asthma between cities of the same country or across countries and (c) studies examining variations in the prevalence of asthma within cities.^19^ Titles and abstracts of the articles identified with the initial search were screened by AR. Full-text papers were retrieved and classified based on the previously mentioned categories. Retrieved texts were evaluated by two reviewers (AR and PC) and a final decision on their inclusion or exclusion was made based on the criteria previously outlined. In case of any doubts and uncertainties, a third author was consulted (LR). Non-systematic review papers and letters to the editor were included to provide a general overview of the topic and as a reference source only and did not provide primary data. A flow chart of the selection process is shown in figure 1.

### Data extraction

A working database was designed using SPSS V.20 including relevant characteristics of the publications: authors(s) name, title, publication year, country, region, gross national income, study design, study approach, area description, age range, sample size, indicators of urbanisation, urban area definition, asthma definition, urban–rural asthma prevalence, unadjusted OR and P value for the urban–rural difference. For studies using more than one category for urban or rural settings (eg, urban and periurban, or rural and perirural), those categories were grouped into either urban or rural area as appropriate.

### Study quality assessment

Study quality was assessed using Strengthening the Reporting of Observational Studies in Epidemiology guidelines,^20^ and ‘Critical Appraisal of Health Research Literature: Prevalence or Incidence of a Health Problem’.^21^ Seven criteria were considered (setting description, population description, sample method, sample size, urban definition, asthma definition and adequate response rate) to classify study quality as high, medium or low. High-quality studies were those providing complete information for these criteria while medium quality studies lacked information for one criterion. Studies lacking information on more than one criterion were considered to be of low quality.

### Statistical analysis

A descriptive analysis was done based on the relevant characteristics of included publications. For cross-sectional studies comparing urban and rural areas, forest plots and unadjusted ORs were used to explore the association between asthma prevalence and area of residence. Because of the large degree of heterogeneity, studies were analysed by asthma definition. A single descriptive pooled OR (and 95% CI) was estimated for each definition using a random-effects model as a synthesis of available information. Results of individual studies were entered into the Cochrane Collaboration Review Manager V.5 and analysed using Metaview V.5. The I^2^ test was used to evaluate heterogeneity between studies. Funnel plots were used to detect bias or systematic heterogeneity by asthma definition groups.

## Results

### Literature search

From 1156 titles and abstracts identified for eligibility in the three databases, 153 articles were selected for a full text review. Seventy articles met our inclusion criteria after full-text review (figure 1). We found two manuscripts with information for two locations in the same publication,^22 23^ and two publications comparing asthma studies in the same location but at two different times.^10 11^ These articles were included in our analysis considering each location (survey) as an independent study. We identified eleven asthma studies that used several categories to define urban and rural - these were re-categorised into a dichotomous urban versus rural classification for inclusion (online supplementary table 1).^10 24–33^ Although we did not consider non-systematic review articles for data extraction, eleven non-systematic review articles addressing the relationship between urbanisation and asthma in LMICs were identified in the literature search.^5 7 8 34–42^ Eighty-three articles were excluded because they were conducted in HICs, studies that lacked data to estimate ORs and duplicate publications.

10.1136/thoraxjnl-2018-211793.supp2 Supplementary data

### Narrative analysis

**Figure 2:**
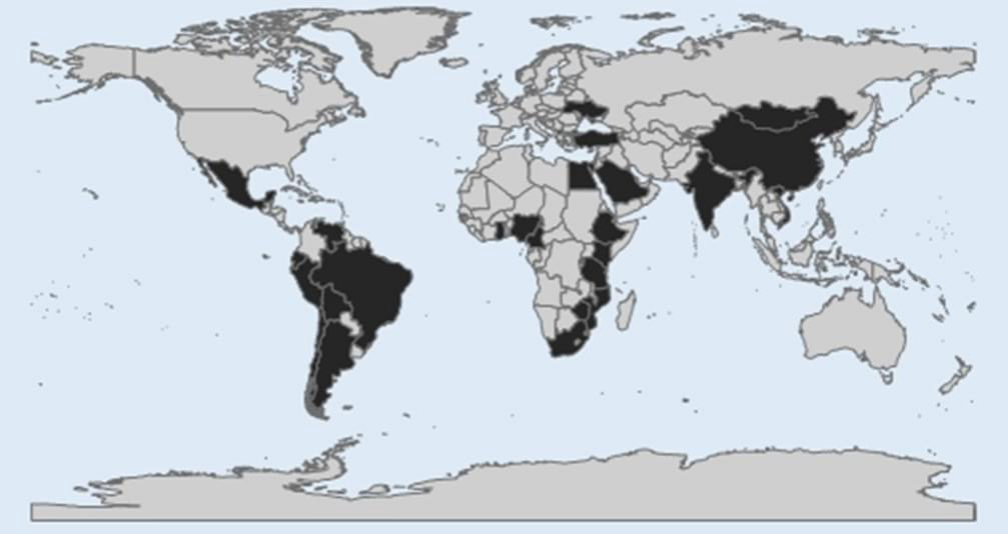
Map of countries in which studies on asthma and urbanisation have been done (countries in black).

Seventy articles published between 1979 and 2017 met the inclusion criteria (table 1). Sixty-three publications compared asthma prevalence between urban and rural areas, five compared asthma prevalence between cities or rural settlements of the same country or among countries and two studied intraurban variations in asthma prevalence. Latin America (LA), Africa and Asia presented a similar number of publications (n=22, n=23, n=24, respectively) (figure 2). Current wheeze was the most used asthma definition (44 publications). Fifty-two publications studied age groups ≤18 years including studies of children (0–12 years), adolescents (12–18 years) or both (0–18 years).

**Table 1:** Characteristics of publications included in the systematic review

Variables and categories	n (%)
Study approach	
Comparing urban vs rural areas	59 (89)
Comparing cities or settlements	5 (8)
Comparing intraurban variation	2 (3)
Region	
Asia	22 (33)
Africa	22 (33)
Latin America	21 (32)
Easter Europe	1 (2)
Study design	
Cross-sectional	58 (88)
Ecologic	7 (10)
Cohort	1 (2)
Methodology	
ISAAC	34 (52)
Other	32 (48)
Asthma definition*	
Wheezing ever	19 (16)
Current wheeze	42 (35)
Doctor diagnosis	24 (20)
Exercise challenge test	10 (8)
Self-report asthma	15 (12)
Questionnaire diagnosis	11 (9)
Age category (years)	
Children (0–12)	15 (23)
Adolescent^12–18^	13 (18)
Children and adolescent (0–18)	21 (34)
Adult (>18)	9 (14)
All ages	7 (11)
Year of the publication	
Before 1990	2 (3)
1990–1999	6 (9)
2000–2009	23 (35)
2010–2017	35 (53)

*Some studies used two or three asthma definitions, so percentages were calculated using the total number of definitions as denominator.

ISAAC, International Study of Asthma and Allergies in Childhood.

### Asthma studies comparing rural and urban areas

**Figure 3:**
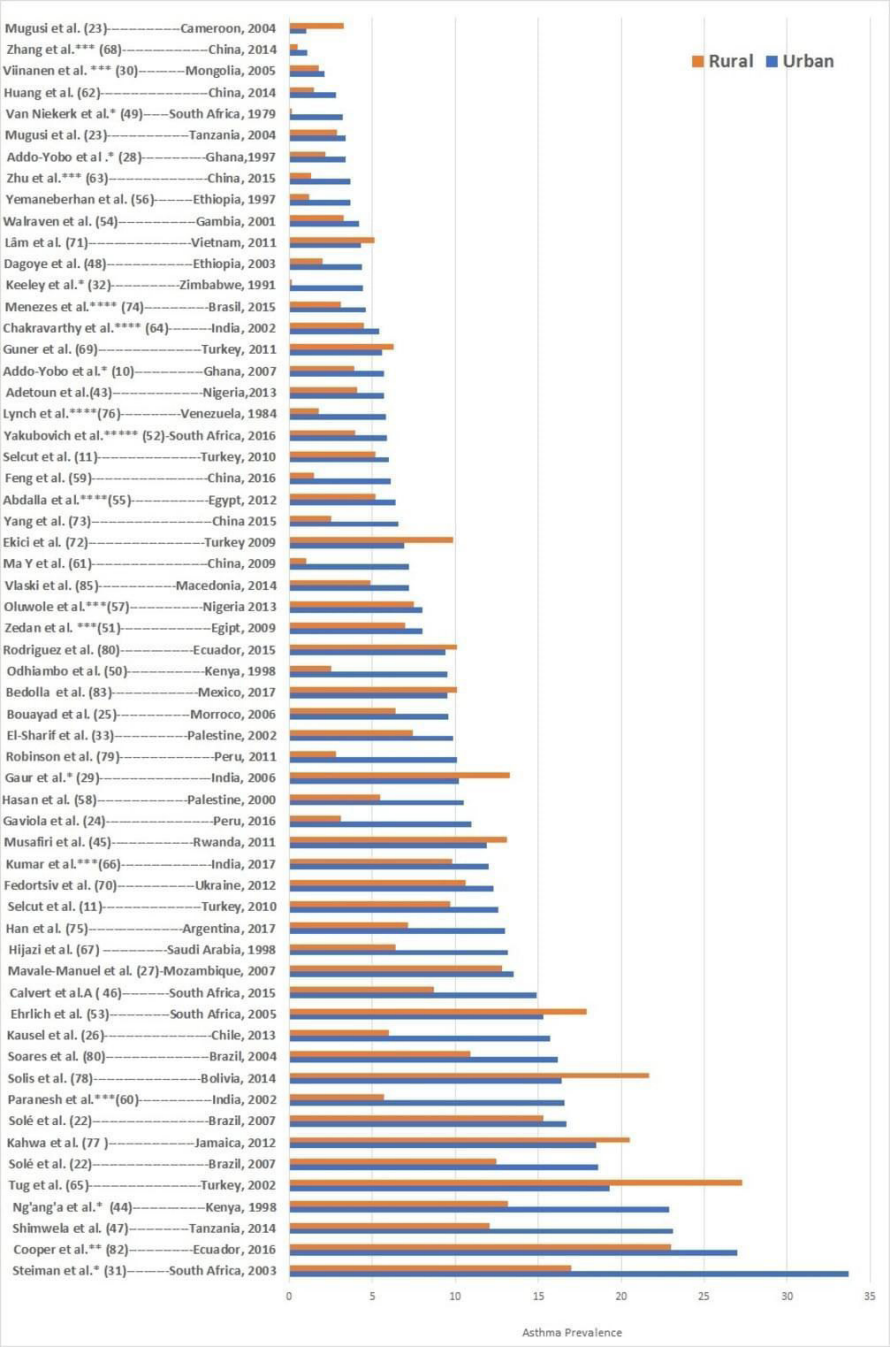
Urban–rural gradient in asthma prevalence in low-income and middle-income countries. asthma definition: (*) exercise challenge test, (**) wheeze ever, (***) asthma questionnaire, (****) doctor diagnosis. All other studies were defined using current wheeze.

**Figure 4:**
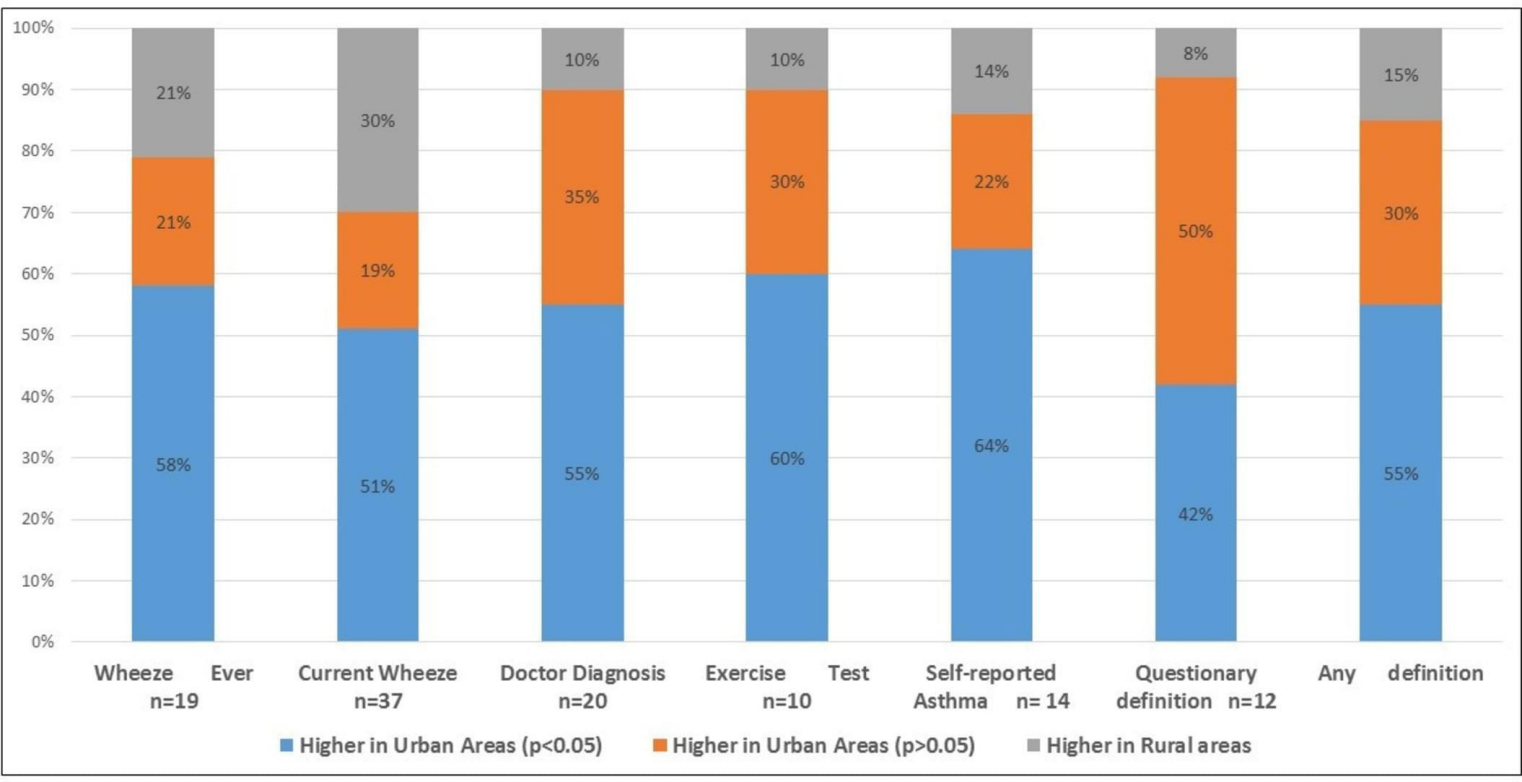
Proportions of studies showing greater prevalence of asthma in urban compared with rural areas by asthma definition.

We found 58 cross-sectional studies, 4 case–control studies conducted in 32 different countries of Africa,^10 23 25 27 28 31 32 43–57^ Asia,^11 29 30 33 58–73^ LA^22 24 26 74–91^ and Eastern Europe.^92^ Figure 3 shows differences in asthma prevalence between urban and rural areas of these countries. Asthma prevalence was generally higher in urban areas. However, proportions of studies showing greater prevalence in urban compared with rural areas varied by asthma definition (figure 4): current wheeze 19/37 studies were statistically significant, wheezing ever 11/19 studies were significant, doctor diagnosis 11/20 studies were significant, exercise challenge test 6/10 studies were significant, self-reported asthma 9/14 were significant and questionnaire diagnosis 5/12 of which five were significant. Complete data are shown in online supplementary table 2.

**Figure 5:**
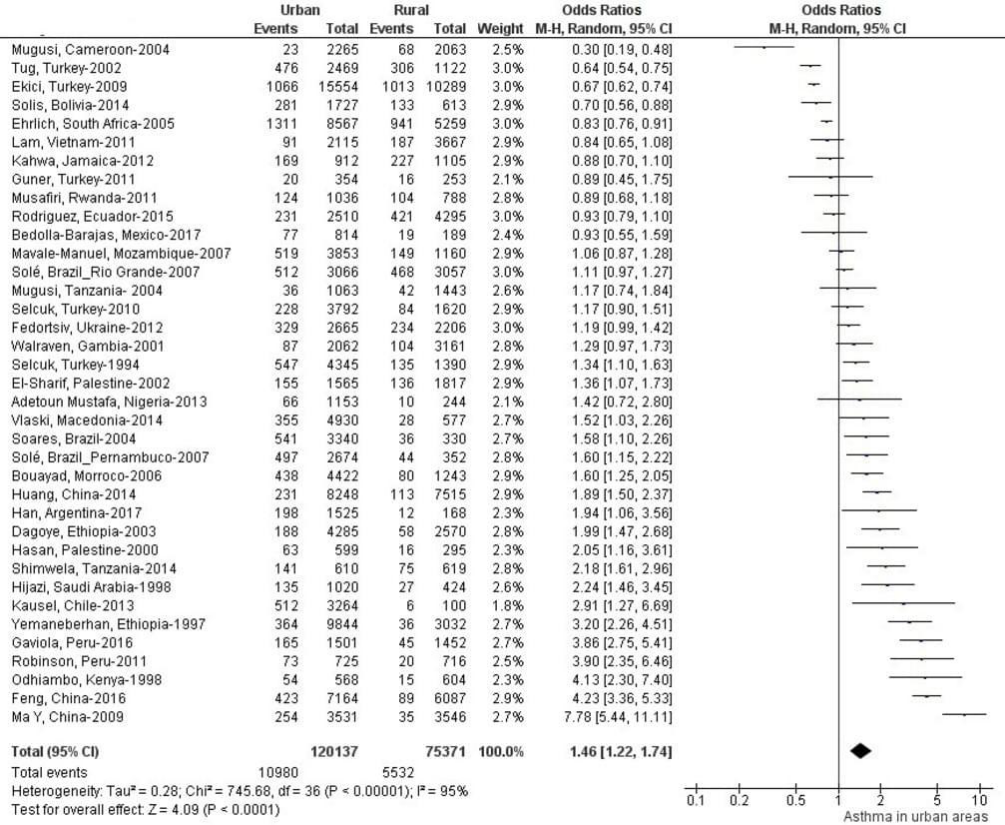
Forest plot and unadjusted ORs for studies using current wheeze to define asthma comparing populations living in urban versus rural areas.

**Figure 6:**
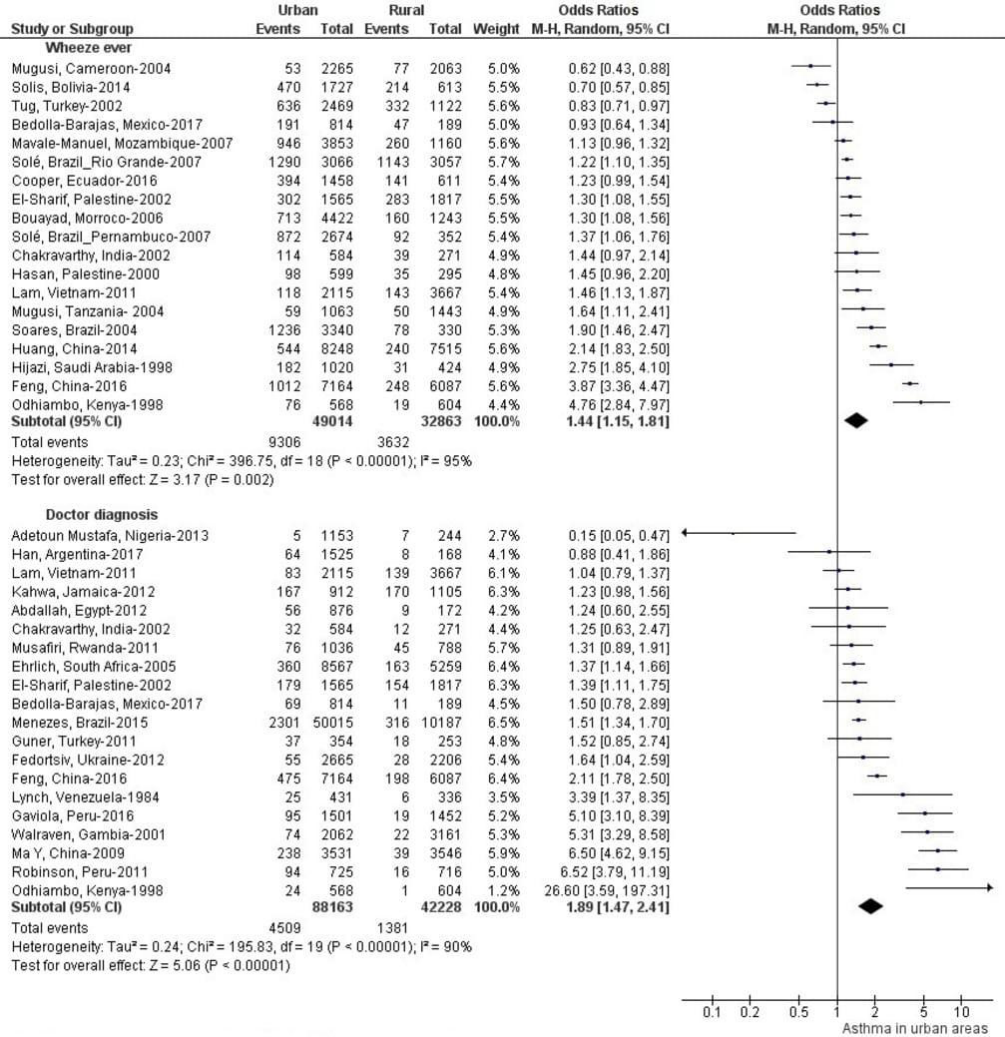
Forest plots and unadjusted ORs for studies using wheezing ever and doctor diagnosis to define asthma comparing populations living in urban versus rural areas.

**Figure 7:**
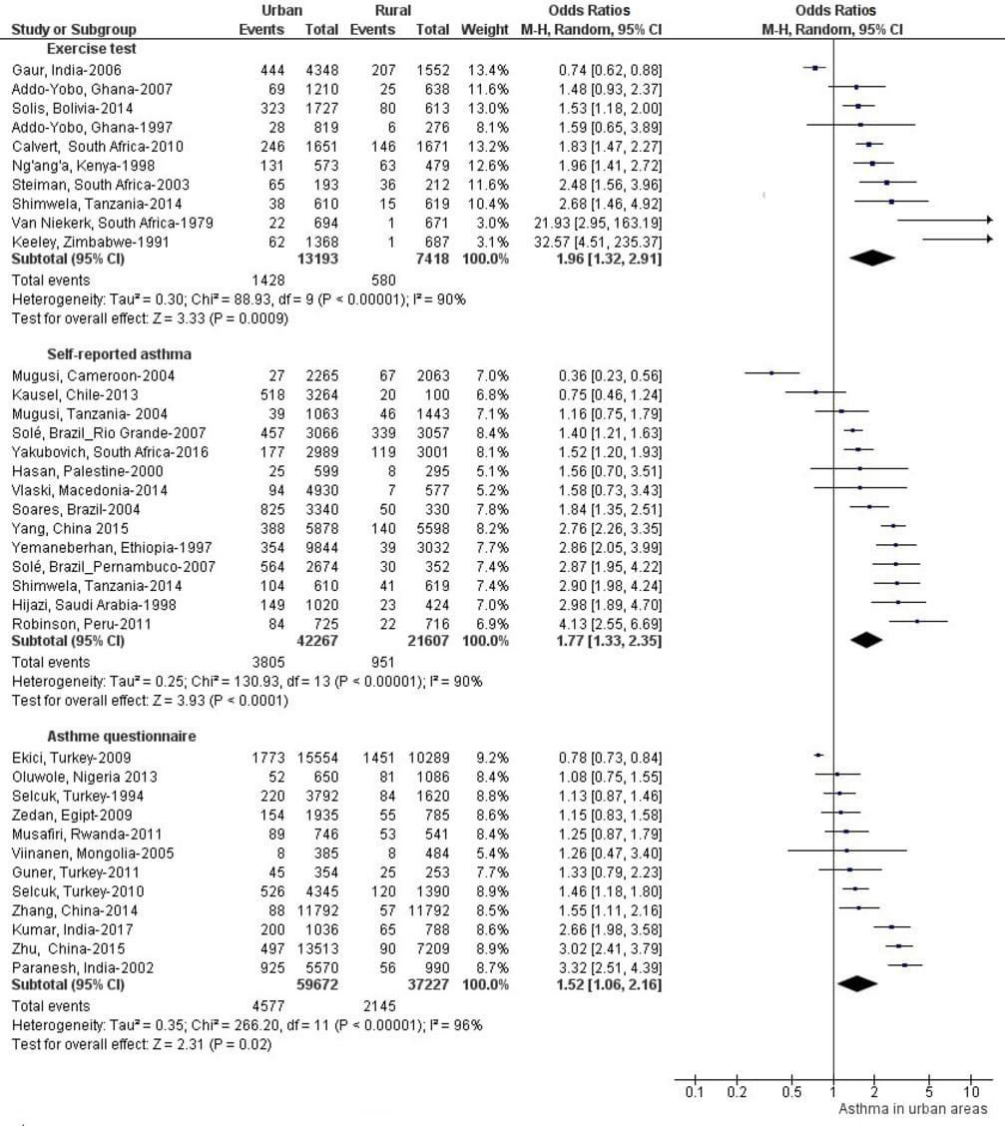
Forest plots and unadjusted ORs for studies using exercise challenge test, self-reported asthma and asthma questionnaire to define asthma comparing populations living in urban versus rural areas.

Pooled unadjusted ORs and forest plots for urban versus rural comparisons of asthma prevalence by asthma definition are shown in figures 5–7. Pooled ORs were: current wheeze, OR: 1.46 (95% CI 1.22 to 1.74); doctor diagnosis, OR: 1.89 (95% CI 1.47 to 2.41); wheeze ever, OR: 1.44 (95% CI 1.15 to 1.81); self-reported asthma, OR: 1.77 (95% CI 1.33 to 2.35); questionnaire-defined asthma, OR: 1.52 (95% CI 1.06 to 2.16); and exercise-induced asthma OR: 1.96 (95% CI 1.32 to 2.91). A high statistical heterogeneity was found (I^2^ >60) for all definitions. Additionally, we calculated pooled unadjusted ORs and forest plots for urban versus rural comparisons of asthma prevalence by age groups, list of countries by national gross income and regions. (Data is shown in online supplementary figures 3-6). Pooled ORs were: age group 0–12 years, OR 1.70 (95% CI 1.37 to 2.11); age group 13–18, OR: 2.09 (95% CI 1.49 to 2.93); low-income countries: OR: 1.48 (95% CI 1.13 to 1.93); lower-middle-income countries, OR: 1.41 (95% CI 1.06 to 1.88); and upper-middle-income countries, OR: 1.70 (95% CI 1.34 to 2.15); Africa, OR 1.56 (95% CI 1.24 to 1.95), Asia, OR 1.62 (95% CI 1.19 to 2.20) and LA, OR 1.52 (95% CI 1.22 to 1.90).

### Asthma studies comparing cities

Publications in this group used ecological designs to compare different urban characteristics between cities of the same country or across countries to infer effects of the urban environment on asthma (table 2). However, this approach was also used to compare other types of settlements as rural communities with other rural communities. In general, five studies were included in this group. The first evaluated associations between asthma prevalence and 11 health and socioeconomic indicators in 20 Brazilian cities and showed that indicators related to urban poverty and inequality were associated with a greater asthma prevalence.^85^ The second evaluated 59 rural communities in Ecuador and correlated community asthma prevalence with different indices constructed to represent the process of urbanisation in the communities.^86^ The study showed that greater levels of urbanisation, particularly with respect to lifestyle and socioeconomic indices, were positively associated with asthma prevalence. The third compared the prevalence of asthma between 31 urban centres across LA using several socioeconomic and environmental indicators.^87^ This study found that social inequalities between cities could be a central determinant of the geographical variation in asthma prevalence within LA. A fourth study conducted in Brazil used 266 municipalities with more than 100 000 inhabitants as the unit of analysis.^88^ This study correlated indicators of socioeconomic factors and violence with the rate of hospital admissions for asthma. The study found a direct correlation between indicators of violence and rates of admission due to asthma, and an inverse correlation with indicators of development. The final study evaluated the effect of urbanisation on hospital admissions and death rates from asthma in 5505 municipalities in Brazil using time series analysis in which urbanisation was defined as the proportion of people living in urban areas by municipality.^89^ The study showed that urban population growth by municipality was associated with a rise in hospital admissions and death rates from asthma in children and young adults.^89^

**Table 2:** Publications comparing asthma prevalence among cities and publication comparing intraurban variation in asthma within cities

Publication	Methodology	Asthma definition	Urban indicators	Findings
Cunha *et al*, 2007 85	Approach: Comparing cities Region/country: Brazil Population: 6–7 and 13–14 years Unit of analyses: cities Sample: 20	Current wheezing	**Socioeconomic indicators**: Illiteracy rate, poverty rate, income, water supply, sanitation, GINI, HDI. **Health indicators**: Infant Mortality, Mortality for external Causes and Hospitals beds.	Asthma prevalence increased with poorer sanitation and with higher infant mortality, GINI index and external mortality. Poverty and inequality seems to be related with asthma prevalence in urban areas of Brazil.
Rodriguez *et al*, 2011 86	Approach: Comparing cities Region/country: Ecuador Population: 5–15 years Unit of analyses: Rural communities Sample: 59	Current wheezing	**Infrastructure Index:** Administrative grade, spatial organisation, transport access, electrical grid, pipe water system, telephone system, health centre, educational institutions. **Socioeconomic Index**: Parent’s education, household income, material goods, access to urban services, housing materials, motors vehicle. **Lifestyles Index:** Junk food consumption, physical exercise, TV viewing, farm activities, pets in house, migration and parasite infections.	Lifestyle and socioeconomic indicators had stronger overall effects on asthma prevalence than infrastructure indicators. Higher asthma prevalence was present in communities with a higher socioeconomic level and a more urbanised lifestyle.
Fattore *et al*, 2014 87	Approach: Comparing cities Region/country: Latin America Population: 6–7 years Unit of analyses: Cities Sample: 31	Current wheezing	**Socioeconomic indicators:** GINI Index and HDI. **Environmental variables**: Water supply, sanitation, crowding. **Health indicators**: Infant mortality and homicide mortality rate.	Income inequality, lack of adequate sanitation, less crowding households, greater reduction in the infant mortality rates and high homicide rates were determinants of asthma symptoms in Latin American urban children.
Tabalipa *et al*,2015 88	Approach: Comparing cities Region/country: Brazil Population: 6–7 and 13–14 years Unit of analyses: Municipalities Sample: 266	Hospital rate admissions (doctor diagnosis)	-**Index of Youth Vulnerability to Violence**: Injury from external causes, incidences of homicides, traffic accidents, education, involvement in crime, poverty and unemployment.	Direct correlation between indicators of violence and rates of admission due to asthma, and an inverse correlation with indicators of development.
Ponte *et al*,2016 89	Approach: Comparing cities Region/country: Brazil Population: 5–24 and 25–39 years Unit of analyses: Municipality Sample: 5505	Hospital rate admissions (doctor diagnosis)	**Socioeconomic indicators:** Per capita income, proportion of the population living in an urban area. **Health indicators:** Number of physicians, number of hospital beds, rate of hospital admission from influenza, access to inhaled corticosteroid for asthma.	An increase in urban population by municipality was associated with lower odds for reduced hospital admissions and death rates from asthma in children and young adults.
Antunes *et al*, 2014 90	Approach: Intraurban variation Region/country: Bahía, Brazil Population: All population Unit of analyses: Census Wards Sample: 93	Hospital rate admissions (doctor diagnosis)	**Socioeconomic indicators**: Income, education, household crowding, presence of slums, GINI Index, sanitation, garbage collection.	Areas of Salvador whose population had lower levels of education and income had higher risk of hospitalisation for respiratory diseases, particularly for asthma and pneumonia.
Dias *et al*, 2016 91	Approach: Intraurban variation Region/country: Belo Horizonte, Brazil Population: 0–14 Unit of analyses: Census Wards Sample	Hospital rate admissions (doctor diagnosis)	-**Health Vulnerability Index**: Inadequate water supply, sanitary sewage and inadequate garbage collection, housing, illiterate population, per capita income, race and ethnicity.	Hospital admissions for asthma were higher in areas of greater social vulnerability, suggesting that social and environmental factors may be determinants of variation in asthma prevalence in urban areas.

GINI, Measure of Inequality; HDI, Human Development Index.

### Asthma studies examining intraurban variations within cities

We found two studies describing the spatial distribution of asthma and their relationships with social and health determinants in two Brazilian cities.^90 91^ Both publications were ecological studies using census wards as the unit of analysis evaluating how living in a particular spatial setting within a city might be associated with asthma. The first study found that areas of Salvador whose population had lower levels of education and income, had a higher risk of hospitalisation for respiratory diseases, particularly for asthma and pneumonia.^90^ The second study conducted in Belo Horizonte found that hospital admissions for asthma were higher in areas of greater social vulnerability, suggesting that social and environmental factors may be determinants of variations in asthma prevalence.^91^

### Study quality

Information on study quality is provided in online supplementary table 3. There was considerable variation in methodological quality between studies. Of the 66 studies included in this systematic review, 26 were considered of low methodological quality. Although most studies used schools as the unit of analysis (comparing urban and rural schoolchildren), the methods by which schools were selected were variable and generally not random but based on convenience samples (n=16). Twenty-three studies provided no information on response rates. Most studies used population size and administrative criteria to define urban and rural areas, comparing populations living in cities with those in rural towns or cities versus communities or villages. However, fifteen studies did not provide general information about the settings in which they were done (n=12). For studies comparing urban and rural areas, sample sizes ranged between 405 and 60 000 subjects. In the case of ecological studies sample size ranged between 20 and 5505 units of analysis.

## Discussion

In this systematic review, we assessed how epidemiological studies conducted in LMICs have addressed the relationship between urbanisation and asthma. We compared also the reported prevalence of asthma in the urban and rural settings studied. Our analyses showed that almost all publications addressing the relationship between asthma and urbanisation come from studies comparing asthma prevalence between urban and rural populations. Few studies from LMICs have used more complex approaches to assess the relationship between urbanisation and asthma. This review provides evidence for an urban–rural gradient in asthma prevalence in LMICs, showing that the risk of asthma is higher in urban compared with rural areas, findings that were consistent irrespective of the asthma definition used. However, any interpretation of these data needs to be cautious because of the high level of heterogeneity between studies.

The study of urbanisation in asthma research has used different methodological approaches to measure the effects of urban areas and urban environments on asthma occurrence, of which the most widely used is comparison urban and rural populations. Although this approach have been useful to identify differences between environmental and social factors that could explain the urban–rural gradient in asthma prevalence,^6 93^ they have limited usefulness understanding the multidimensional nature of urbanisation. Issues such as diverse dimensions of urban environments, differences in lifestyle between populations, distinct levels of urbanisation between urban centres and changes over time, cannot be properly addressed using this approach. For example, in our review, 13 studies reported a similar or a higher prevalence of asthma in rural compared with urban areas. It is likely that differences in lifestyle between urban and rural population may be responsible for these findings. Indeed, a non-systematic review of urban–rural comparisons of asthma prevalence showed only minimal differences, particularly where socioeconomic and environmental factors were comparable between urban and rural populations.^42^ Thus, rural and urban populations that share similar living conditions and socioeconomic factors are likely to have comparable asthma risks. Such a situation is commonly found in HICs where rural and urban populations have similar lifestyles and standards of living, but also in LMICs where many urban (and periurban) localities may have similar living conditions to more rural settings, and in the case of urban slums living conditions may be worse than many rural settings.^94^ This is important because of the frequent misconception in asthma studies that urban populations in LMICs live in cleaner and healthier environment.^95^

A second common approach has been to compare asthma prevalence or asthma hospitalisation rates by different urban characteristics of cities, municipalities or communities—such as infrastructure, socioeconomic indicators, level of violence, urban services, health indicators, among others—to identify features of the urbanisation process that could be related to asthma prevalence. In studies comparing cities, a higher prevalence of asthma was observed in those cities with poor sanitation, high infant mortality, social inequalities and elevated levels of violence. Overall, these studies indicate that social deprivation in cities could contribute to asthma risk. In agreement with this, cross-sectional studies from the USAnited States and LA have observed associations between asthma risk and poverty and lack of basic services in urban areas.^42 96 97^ In the Ecuadorian study comparing rural communities, indices representing different domains of the urbanisation process as socioeconomic, lifestyle, urban infrastructure and a summary urbanisation derived from representative variables of each of these, were associated with asthma prevalence. While significant heterogeneity was observed in the level of urbanisation between rural communities, the community prevalence of asthma increased with greater levels of urbanisation, especially with indices representing lifestyle and socioeconomic factors. These findings mirror those of other studies done in LMICs.^24 26 30 33^ For example, a cross-sectional study from Mongolia compared the prevalence of asthma and RAD in localities with different levels of urbanisation: city, urban town and villages. The study showed an increasing prevalence of allergic diseases with greater level of urbanisation.^30^ It is important to highlight that comparing cities (or other urban areas) in the same country offer a relative solution to the lack of a general definition of urbanisation present in asthma studies comparing urban and rural areas from diverse parts of the world. The city comparison approach within the same country is based on (1) urban area definition is the same for all settlements, and (2) urban characteristics of the cities are more comparable within a country than between countries, especially because factors such as climate, culture and other characteristic are likely to be similar. Likewise, comparisons between rural localities allow the study of urbanisation processes and *urban sprawl* in transitional societies where changes in lifestyle and environmental factors occur more rapidly. A weakness of studies using cities or settlements as the unit of analysis is the assumption that aggregate behaviours or characteristics at the city level are equally important for all residents. This ecologic fallacy requires a cautious interpretation of findings from such studies.^98^

Intraurban studies evaluate how living in a particular area of a city may be associated with asthma outcomes. Such studies tend to use spatial groupings of individuals, commonly represented by neighbourhoods or census wards, to assess the effect of place of residence within an urban area on community or individual health.^19^ These studies often require spatial and socioeconomic information in these localities at individual and contextual levels, commonly provided by censuses and other publicly available data sources. For asthma research, this approach would be appropriate for addressing questions related to identifying the characteristics of areas within cities that may be associated with asthma. However, few such studies have been done in LMICs.

### Limitations of this review

Studies evaluating specific characteristics of the urban environment, such as air pollution or distance to an urban location,^99^ were not included. In the case of air pollution, there is a large literature and this topic may be better dealt with separately. We considered only studies done in LMICs because these countries share historical and developmental processes determining the evolution of the urban environment that are distinct from those that have occurred in HICs.^100^ Other ecological studies, especially those related to the International Study of Asthma and Allergies in Childhood, were not included here because they use populations from both LMICs and HICs.^101–103^ Because of the large degree of heterogeneity between studies (different study setting, population age, asthma definitions, urban–rural definitions) and variable methodological quality, pooled ORs estimated by asthma definition need to be interpreted with caution. Finally, although we carried out a thorough search of the literature and produced funnel plots to investigate potential publication bias (see online supplementary figure 2), the plots were not suggestive of publication bias, but we cannot completely exclude the possibility that studies that do not show a positive association are less likely to be published.

## Conclusions

This systematic review analysed the effects from the published literature of urbanisation on the prevalence of asthma in LMICs. Published epidemiological studies addressing this issue have mostly used one of three methodological approaches; comparisons of asthma prevalence between urban and rural areas, comparisons of cities within and between countries, and comparisons of areas within cities. Similarly, published studies have used a variety of definitions to define asthma. However, despite such heterogeneity in asthma definitions a number of consistent patterns emerged in this systematic review: (1) irrespective of the asthma definitions used, the prevalence of asthma was greater in urban than rural areas in most but not all studies; (2) indicators of social deprivation, inequality and or poverty within or between cities were associated with the prevalence of asthma or hospitalisation rates for asthma; and (3) even at the rural level, indicators of urbanisation, particularly lifestyle and socioeconomic factors, were associated with asthma prevalence. Overall, these findings provide evidence that urban residence and urbanisation are important determinants of asthma prevalence but do not permit us to identify which aspects of the urbanisation process are most important as determinants of risk due to most of the studies exploring the effects of urbanisation on asthma have used the simple urban–rural approach. Such method does not allow us to consider the multifactorial dimensions of the urbanisation process and cannot identify specific factors or conditions associated with asthma risk. We need to start thinking about more complex chains of causation in urban studies and asthma. An important issue for studies of the effects of urbanisation and asthma is a lack of an adequate conceptual model for how social, psychological and biological determinants within urbanisation processes interact to affect asthma risk. A better understanding of how such processes operate is likely to lead to a better understanding of asthma causation and potential strategies to the primary prevention of this important debilitating disease. We believe that studies addressing the multifactorial dimensions of the urbanisation process using the city comparison and the intraurban comparison approaches will help to generate more closely specified causal models which also help to clarify the distinction between confounding and intervening variables. Additionally, there is a clear need for an accurate standardised operational definition of asthma and a clearer and more precise definitions of ‘urbanisation’ and ‘urban areas’. This would facilitate aetiological research, comparisons between locations (especially in international studies) and estimations of asthma prevalence in epidemiological studies.
