# Supplementary material for: Urbanisation and asthma in low-income and middle-income countries: a systematic review of the urban–rural differences in asthma prevalence
Source: Thorax. 2019 Jul 5;74(11):1020–30. doi: 10.1136/thoraxjnl-2018-211793 (PMC6860411; doi:10.1136/thoraxjnl-2018-211793)
Supplement: Supplementary data [file thoraxjnl-2018-211793supp001.pdf]

Supplementary Figures

Supplementary Figure 1. Search terms and equations used for this review in PubMed, Scielo databases.

(asthma[tiab]) OR asthma prevalence*[tiab] OR wheeze*[tiab]
OR allergic diseases[tiab] OR allergic disorders[tiab] OR allerg*[tiab])
AND

(urbanization[tiab] OR urbanisation[tiab] OR urban area[tiab] OR rural area[tiab]
OR urban rural difference[tiab] OR rural urban comparison[tiab] OR urban environment
OR urban population[tiab] OR metropolitan area OR city OR inner-city[tiab]
OR municipal OR urban neighbourhood[tiab] OR country-side OR provincial
OR provincial OR rural community[Mesh]).
OR

(urban indicators[tiab] OR urban poverty OR socioeconomic status
OR urban deprivation OR socioeconomic factors
OR deprived areas OR rural poverty[Mesh])

Rodriguez A, *et al. Thorax* 2019; 0:1020–1030. doi: 10.1136/thoraxjnl-2018-211793

Supplementary Figure 2. Funnel Plots for asthma studies comparing populations living in urban versus rural areas by asthma definitions: (A) current wheeze, (B) wheeze ever, (C) doctor diagnosis, (D) exercise test, (E) self-reported asthma, and (F) asthma questionnaire.

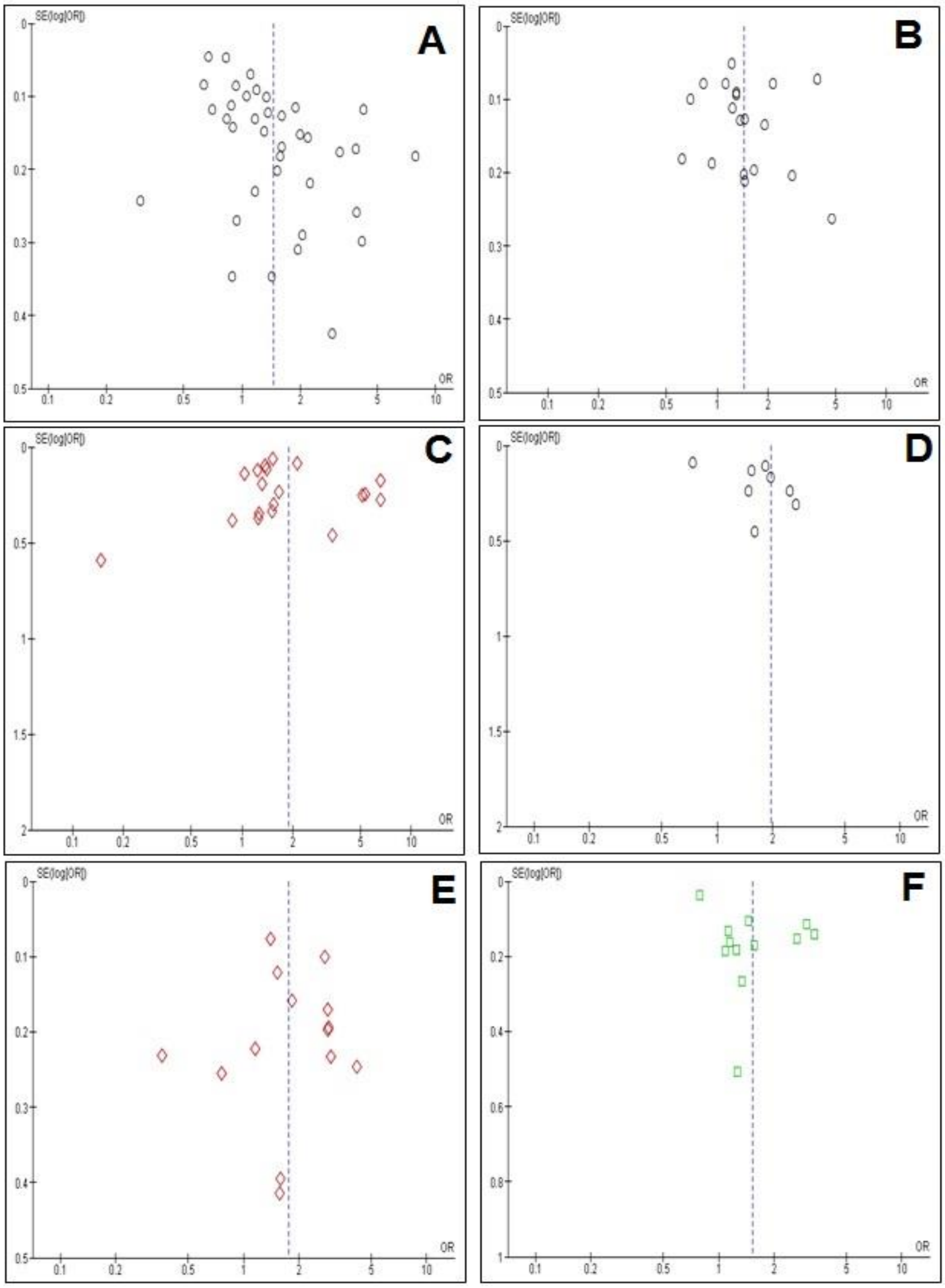

Supplementary Figures 3. Forest Plot and unadjusted Odd Ratios for asthma comparing populations living in urban versus rural areas by populations groups: (A) 0 – 12 years, (B) 12-18 years and (C) 0-18 years.

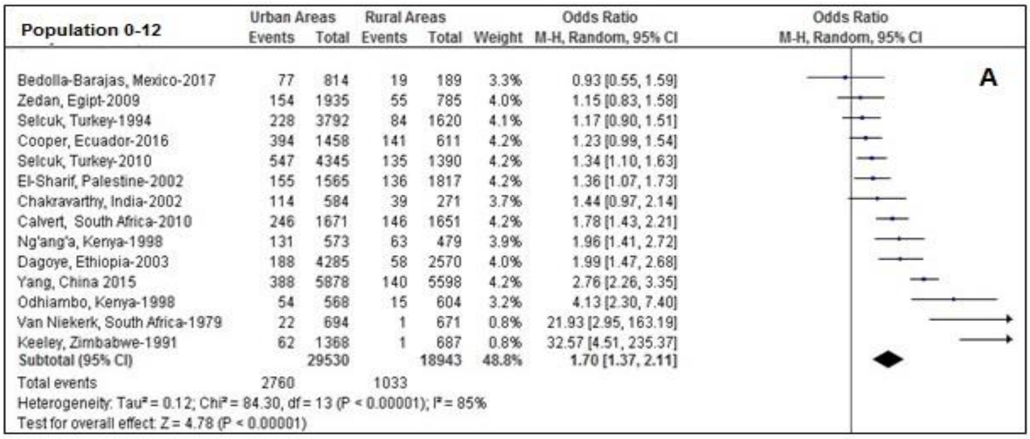

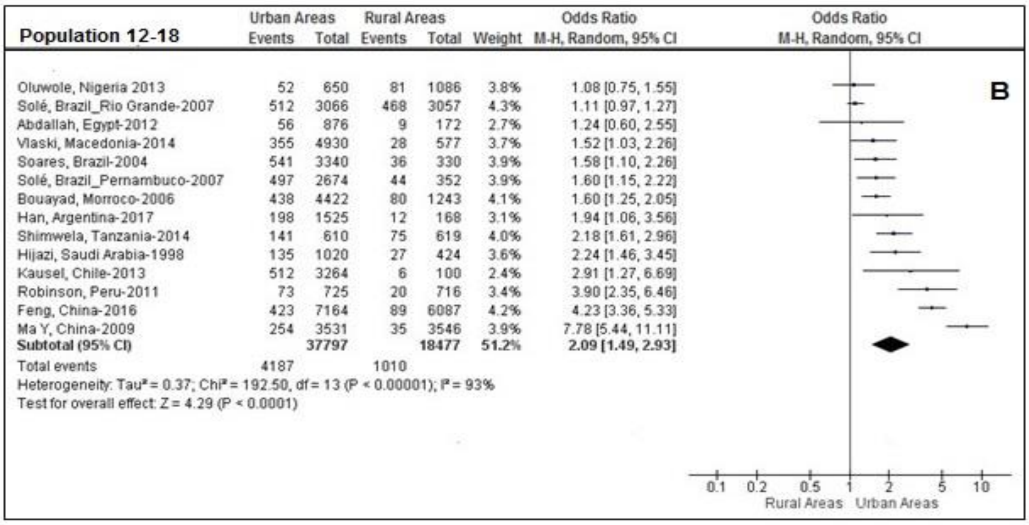

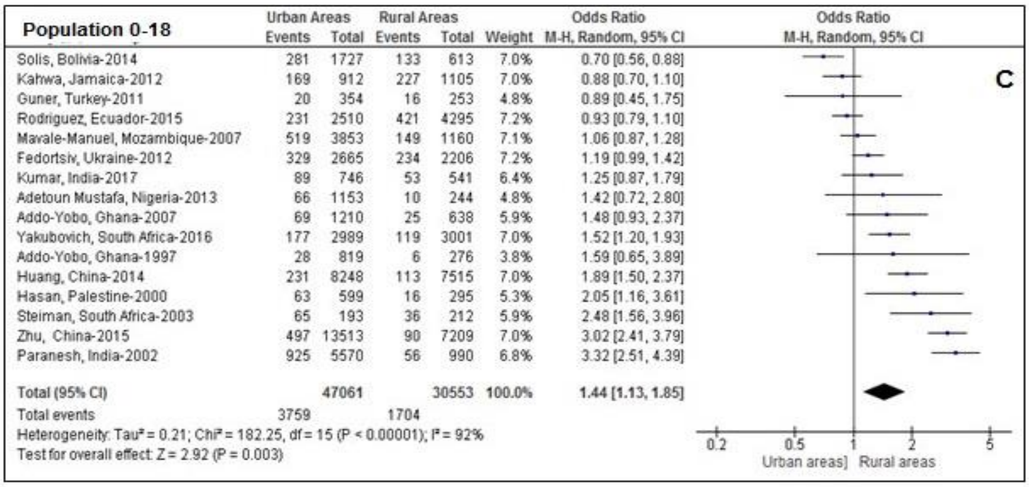

Supplementary Figures 4. Forest Plot and unadjusted Odd Ratios for asthma comparing populations living in urban versus rural areas by populations groups: (D) >18 years and (E) 0-65 years.

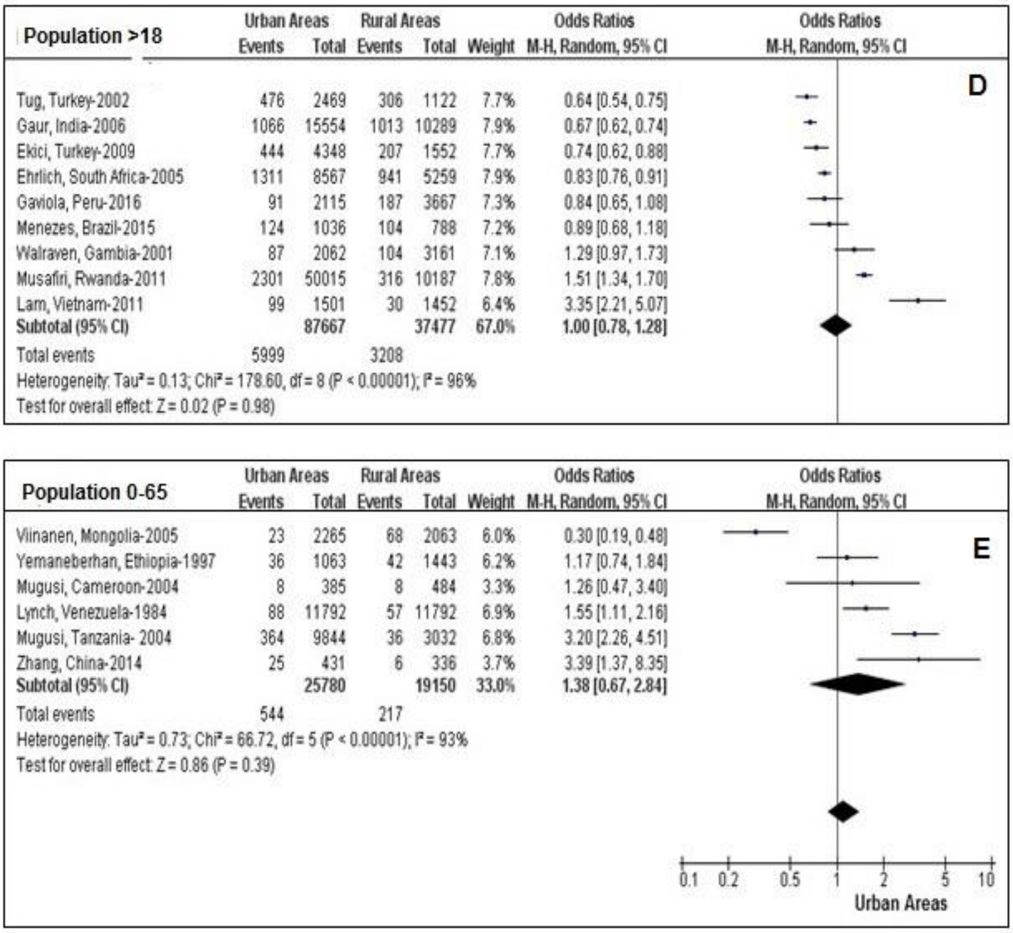

Supplementary Figures 5. Forest Plot and unadjusted Odd Ratios for asthma comparing populations living in urban versus rural areas by gross national income: (A) Low income countries, (B) Low meddle-income countries, and (C) Upper meddle-income countries.

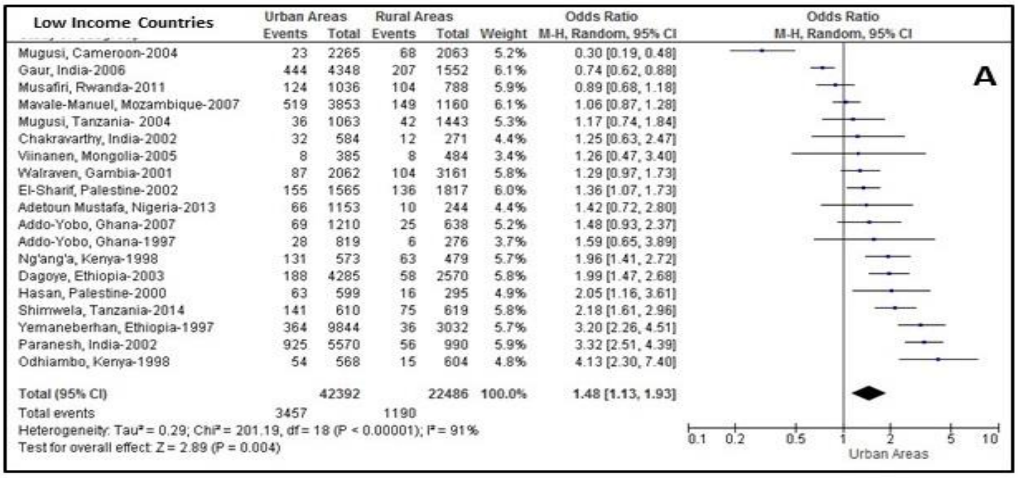

Low Income Countries	Urban Areas Events	Urban Areas Total	Rural Areas Events	Rural Areas Total	Weight	Odds Ratio M-H, Random, 95% CI
Mugusi, Cameroon-2004	23	2265	68	2063	5.2%	0.30 [0.19, 0.48]
Gaur, India-2006	444	4348	207	1552	6.1%	0.74 [0.62, 0.88]
Musafiri, Rwanda-2011	124	1036	104	788	5.9%	0.89 [0.68, 1.18]
Mavale-Manuel, Mozambique-2007	519	3853	149	1160	6.1%	1.06 [0.87, 1.28]
Mugusi, Tanzania- 2004	36	1063	42	1443	5.3%	1.17 [0.74, 1.84]
Chakravarthy, India-2002	32	584	12	271	4.4%	1.25 [0.63, 2.47]
Viinanen, Mongolia-2005	8	385	8	484	3.4%	1.26 [0.47, 3.40]
Walraven, Gambia-2001	87	2062	104	3161	5.8%	1.29 [0.97, 1.73]
El-Sharif, Palestine-2002	155	1565	136	1817	6.0%	1.36 [1.07, 1.73]
Adetoun Mustafa, Nigeria-2013	66	1153	10	244	4.4%	1.42 [0.72, 2.80]
Addo-Yobo, Ghana-2007	69	1210	25	638	5.2%	1.48 [0.93, 2.37]
Addo-Yobo, Ghana-1997	28	819	6	276	3.7%	1.59 [0.65, 3.89]
Ng'ang'a, Kenya-1998	131	573	63	479	5.7%	1.96 [1.41, 2.72]
Dagoye, Ethiopia-2003	188	4285	58	2570	5.8%	1.99 [1.47, 2.68]
Hasan, Palestine-2000	63	599	16	295	4.9%	2.05 [1.16, 3.61]
Shimwela, Tanzania-2014	141	610	75	619	5.8%	2.18 [1.61, 2.96]
Yemaneberhan, Ethiopia-1997	364	9844	36	3032	5.7%	3.20 [2.26, 4.51]
Paranesh, India-2002	925	5570	56	990	5.9%	3.32 [2.51, 4.39]
Odhiambo, Kenya-1998	54	568	15	604	4.8%	4.13 [2.30, 7.40]
**Total (95% CI)**		**42392**		**22486**	**100.0%**	**1.48 [1.13, 1.93]**
Total events	3457		1190			

Heterogeneity: Tau² = 0.29; Chi² = 201.19, df = 18 (P < 0.00001); I² = 91%
Test for overall effect: Z = 2.89 (P = 0.004)

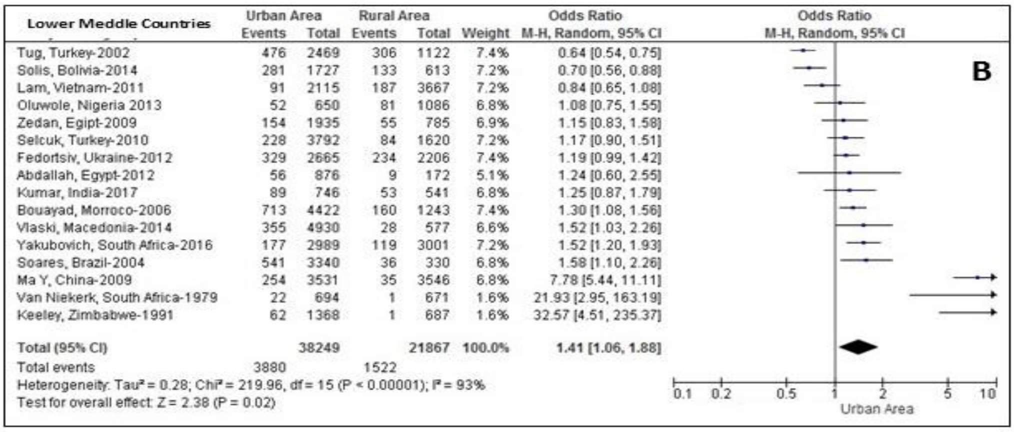

Lower Meddle Countries	Urban Area Events	Urban Area Total	Rural Area Events	Rural Area Total	Weight	Odds Ratio M-H, Random, 95% CI
Tug, Turkey-2002	476	2469	306	1122	7.4%	0.64 [0.54, 0.75]
Solis, Bolivia-2014	281	1727	133	613	7.2%	0.70 [0.56, 0.88]
Lam, Vietnam-2011	91	2115	187	3667	7.2%	0.84 [0.65, 1.08]
Oluwole, Nigeria 2013	52	650	81	1086	6.8%	1.08 [0.75, 1.55]
Zedan, Egipt-2009	154	1935	55	785	6.9%	1.15 [0.83, 1.58]
Selcuk, Turkey-2010	228	3792	84	1620	7.2%	1.17 [0.90, 1.51]
Fedortsiv, Ukraine-2012	329	2665	234	2206	7.4%	1.19 [0.99, 1.42]
Abdallah, Egypt-2012	56	876	9	172	5.1%	1.24 [0.60, 2.55]
Kumar, India-2017	89	746	53	541	6.8%	1.25 [0.87, 1.79]
Bouayad, Morroco-2006	713	4422	160	1243	7.4%	1.30 [1.08, 1.56]
Vlaski, Macedonia-2014	355	4930	28	577	6.6%	1.52 [1.03, 2.26]
Yakubovich, South Africa-2016	177	2989	119	3001	7.2%	1.52 [1.20, 1.93]
Soares, Brazil-2004	541	3340	36	330	6.8%	1.58 [1.10, 2.26]
Ma Y, China-2009	254	3531	35	3546	6.8%	7.78 [5.44, 11.11]
Van Niekerk, South Africa-1979	22	694	1	671	1.6%	21.93 [2.95, 163.19]
Keeley, Zimbabwe-1991	62	1368	1	687	1.6%	32.57 [4.51, 235.37]
**Total (95% CI)**		**38249**		**21867**	**100.0%**	**1.41 [1.06, 1.88]**
Total events	3880		1522			

Heterogeneity: Tau² = 0.28; Chi² = 219.96, df = 15 (P < 0.00001); I² = 93%
Test for overall effect: Z = 2.38 (P = 0.02)

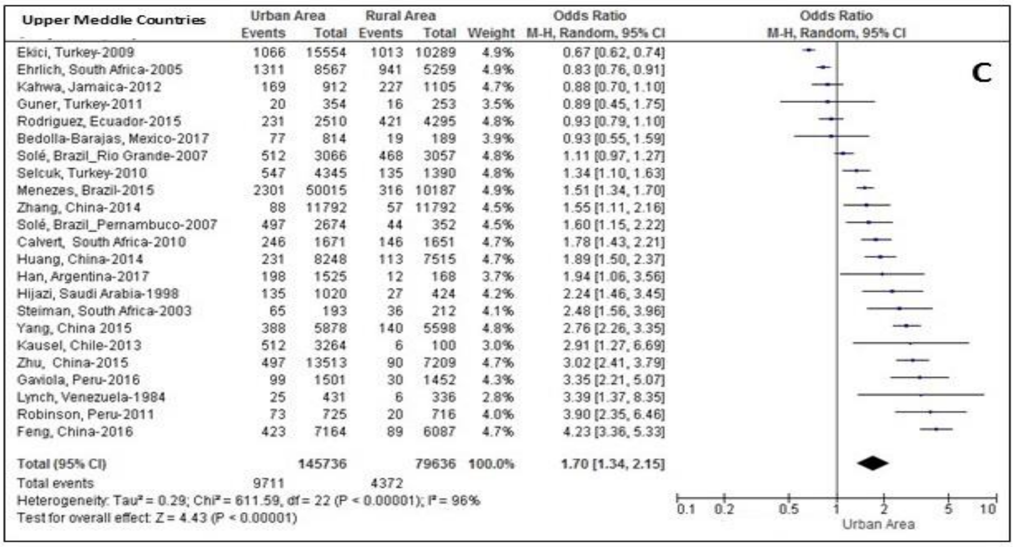

Upper Meddle Countries	Urban Area Events	Urban Area Total	Rural Area Events	Rural Area Total	Weight	Odds Ratio M-H, Random, 95% CI
Ekici, Turkey-2009	1066	15554	1013	10289	4.9%	0.67 [0.62, 0.74]
Ehrlich, South Africa-2005	1311	8567	941	5259	4.9%	0.83 [0.76, 0.91]
Kahwa, Jamaica-2012	169	912	227	1105	4.7%	0.88 [0.70, 1.10]
Guner, Turkey-2011	20	354	16	253	3.5%	0.89 [0.45, 1.75]
Rodriguez, Ecuador-2015	231	2510	421	4295	4.8%	0.93 [0.79, 1.10]
Bedolla-Barajas, Mexico-2017	77	814	19	189	3.9%	0.93 [0.55, 1.59]
Solé, Brazil_Rio Grande-2007	512	3066	468	3057	4.8%	1.11 [0.97, 1.27]
Selcuk, Turkey-2010	547	4345	135	1390	4.8%	1.34 [1.10, 1.63]
Menezes, Brazil-2015	2301	50015	316	10187	4.9%	1.51 [1.34, 1.70]
Zhang, China-2014	88	11792	57	11792	4.5%	1.55 [1.11, 2.16]
Solé, Brazil_Pernambuco-2007	497	2674	44	352	4.5%	1.60 [1.15, 2.22]
Calvert, South Africa-2010	246	1671	146	1651	4.7%	1.78 [1.43, 2.21]
Huang, China-2014	231	8248	113	7515	4.7%	1.89 [1.50, 2.37]
Han, Argentina-2017	198	1525	12	168	3.7%	1.94 [1.06, 3.56]
Hijazi, Saudi Arabia-1998	135	1020	27	424	4.2%	2.24 [1.46, 3.45]
Steiman, South Africa-2003	65	193	36	212	4.1%	2.48 [1.56, 3.96]
Yang, China 2015	388	5878	140	5598	4.8%	2.76 [2.26, 3.35]
Kausel, Chile-2013	512	3264	6	100	3.0%	2.91 [1.27, 6.69]
Zhu, China-2015	497	13513	90	7209	4.7%	3.02 [2.41, 3.79]
Gaviola, Peru-2016	99	1501	30	1452	4.3%	3.35 [2.21, 5.07]
Lynch, Venezuela-1984	25	431	6	336	2.8%	3.39 [1.37, 8.35]
Robinson, Peru-2011	73	725	20	716	4.0%	3.90 [2.35, 6.46]
Feng, China-2016	423	7164	89	6087	4.7%	4.23 [3.36, 5.33]
**Total (95% CI)**		**145736**		**79636**	**100.0%**	**1.70 [1.34, 2.15]**
Total events	9711		4372			

Heterogeneity: Tau² = 0.29; Chi² = 611.59, df = 22 (P < 0.00001); I² = 96%
Test for overall effect: Z = 4.43 (P < 0.00001)

Supplementary Figures 6. Forest Plot and unadjusted Odd Ratios for asthma comparing populations living in urban versus rural areas by region: (A) African countries, (B) Asian countries, and (C) Latin American countries.

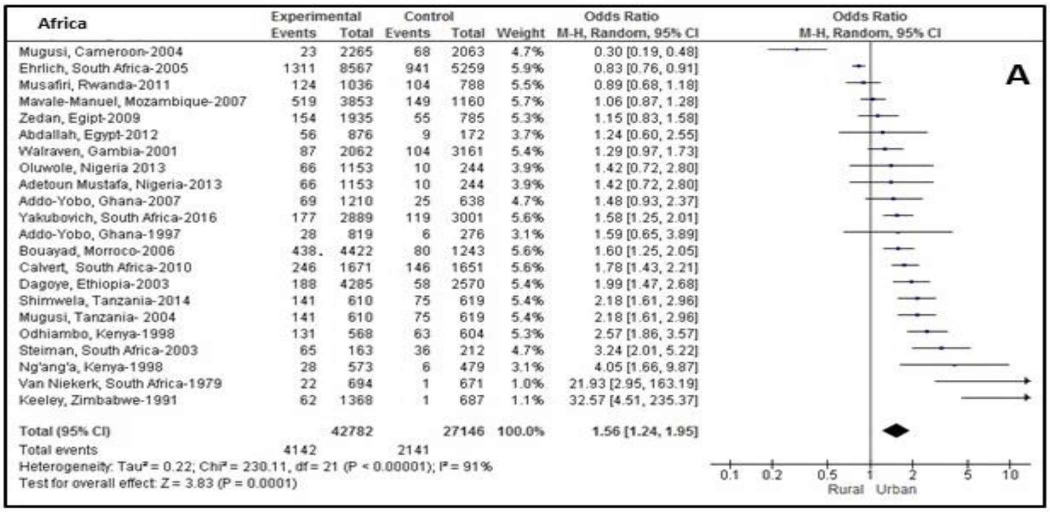

Africa	Experimental Events	Experimental Total	Control Events	Control Total	Weight	Odds Ratio M-H, Random, 95% CI
Mugusi, Cameroon-2004	23	2265	68	2063	4.7%	0.30 [0.19, 0.48]
Ehrlich, South Africa-2005	1311	8567	941	5259	5.9%	0.83 [0.76, 0.91]
Musafiri, Rwanda-2011	124	1036	104	788	5.5%	0.89 [0.68, 1.18]
Mavale-Manuel, Mozambique-2007	519	3853	149	1160	5.7%	1.06 [0.87, 1.28]
Zedan, Egipt-2009	154	1935	55	785	5.3%	1.15 [0.83, 1.58]
Abdallah, Egypt-2012	56	876	9	172	3.7%	1.24 [0.60, 2.55]
Walraven, Gambia-2001	87	2062	104	3161	5.4%	1.29 [0.97, 1.73]
Oluwole, Nigeria 2013	66	1153	10	244	3.9%	1.42 [0.72, 2.80]
Adetoun Mustafa, Nigeria-2013	66	1153	10	244	3.9%	1.42 [0.72, 2.80]
Addo-Yobo, Ghana-2007	69	1210	25	638	4.7%	1.48 [0.93, 2.37]
Yakubovich, South Africa-2016	177	2889	119	3001	5.6%	1.58 [1.25, 2.01]
Addo-Yobo, Ghana-1997	28	819	6	276	3.1%	1.59 [0.65, 3.89]
Bouayad, Morroco-2006	438.	4422	80	1243	5.6%	1.60 [1.25, 2.05]
Calvert, South Africa-2010	246	1671	146	1651	5.6%	1.78 [1.43, 2.21]
Dagoye, Ethiopia-2003	188	4285	58	2570	5.4%	1.99 [1.47, 2.68]
Shimwela, Tanzania-2014	141	610	75	619	5.4%	2.18 [1.61, 2.96]
Mugusi, Tanzania- 2004	141	610	75	619	5.4%	2.18 [1.61, 2.96]
Odhiambo, Kenya-1998	131	568	63	604	5.3%	2.57 [1.86, 3.57]
Steiman, South Africa-2003	65	163	36	212	4.7%	3.24 [2.01, 5.22]
Ng'ang'a, Kenya-1998	28	573	6	479	3.1%	4.05 [1.66, 9.87]
Van Niekerk, South Africa-1979	22	694	1	671	1.0%	21.93 [2.95, 163.19]
Keeley, Zimbabwe-1991	62	1368	1	687	1.1%	32.57 [4.51, 235.37]
**Total (95% CI)**		**42782**		**27146**	**100.0%**	**1.56 [1.24, 1.95]**
Total events	4142		2141			

Heterogeneity: Tau² = 0.22; Chi² = 230.11, df = 21 (P < 0.00001); I² = 91%
Test for overall effect: Z = 3.83 (P = 0.0001)

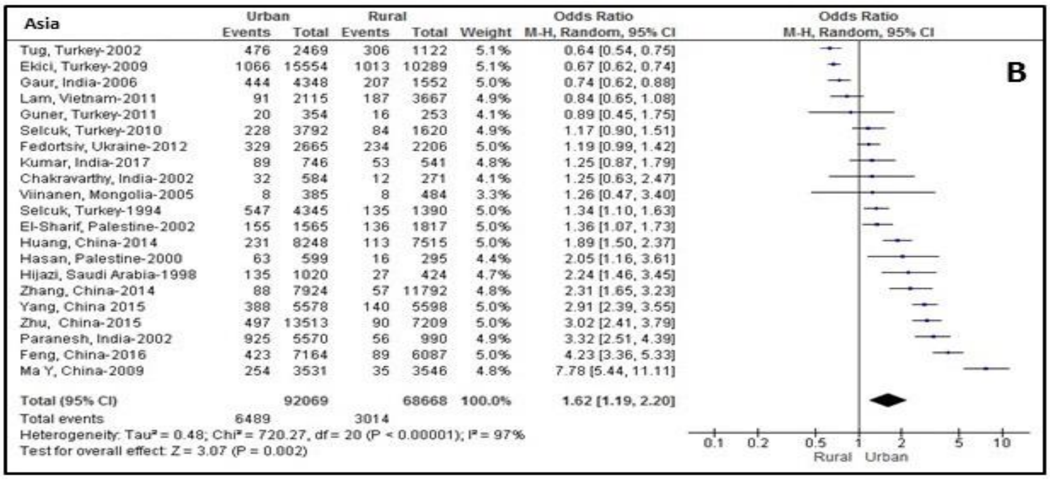

Asia	Urban Events	Urban Total	Rural Events	Rural Total	Weight	Odds Ratio M-H, Random, 95% CI
Tug, Turkey-2002	476	2469	306	1122	5.1%	0.64 [0.54, 0.75]
Ekici, Turkey-2009	1066	15554	1013	10289	5.1%	0.67 [0.62, 0.74]
Gaur, India-2006	444	4348	207	1552	5.0%	0.74 [0.62, 0.88]
Lam, Vietnam-2011	91	2115	187	3667	4.9%	0.84 [0.65, 1.08]
Guner, Turkey-2011	20	354	16	253	4.1%	0.89 [0.45, 1.75]
Selcuk, Turkey-2010	228	3792	84	1620	4.9%	1.17 [0.90, 1.51]
Fedortsiv, Ukraine-2012	329	2665	234	2206	5.0%	1.19 [0.99, 1.42]
Kumar, India-2017	89	746	53	541	4.8%	1.25 [0.87, 1.79]
Chakravarthy, India-2002	32	584	12	271	4.1%	1.25 [0.63, 2.47]
Viinanen, Mongolia-2005	8	385	8	484	3.3%	1.26 [0.47, 3.40]
Selcuk, Turkey-1994	547	4345	135	1390	5.0%	1.34 [1.10, 1.63]
El-Sharif, Palestine-2002	155	1565	136	1817	5.0%	1.36 [1.07, 1.73]
Huang, China-2014	231	8248	113	7515	5.0%	1.89 [1.50, 2.37]
Hasan, Palestine-2000	63	599	16	295	4.4%	2.05 [1.16, 3.61]
Hijazi, Saudi Arabia-1998	135	1020	27	424	4.7%	2.24 [1.46, 3.45]
Zhang, China-2014	88	7924	57	11792	4.8%	2.31 [1.65, 3.23]
Yang, China 2015	388	5578	140	5598	5.0%	2.91 [2.39, 3.55]
Zhu, China-2015	497	13513	90	7209	5.0%	3.02 [2.41, 3.79]
Paranesh, India-2002	925	5570	56	990	4.9%	3.32 [2.51, 4.39]
Feng, China-2016	423	7164	89	6087	5.0%	4.23 [3.36, 5.33]
Ma Y, China-2009	254	3531	35	3546	4.8%	7.78 [5.44, 11.11]
**Total (95% CI)**		**92069**		**68668**	**100.0%**	**1.62 [1.19, 2.20]**
Total events	6489		3014			

Heterogeneity: Tau² = 0.48; Chi² = 720.27, df = 20 (P < 0.00001); I² = 97%
Test for overall effect: Z = 3.07 (P = 0.002)

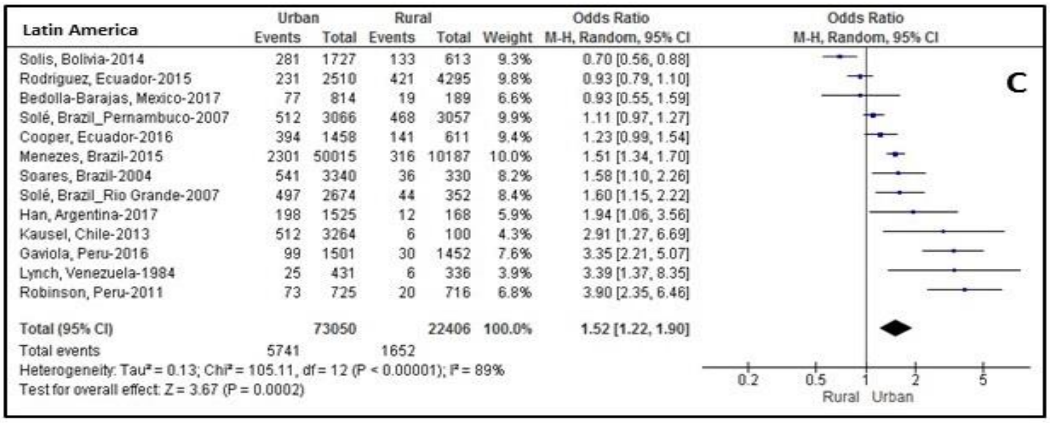

Latin America	Urban Events	Urban Total	Rural Events	Rural Total	Weight	Odds Ratio M-H, Random, 95% CI
Solis, Bolivia-2014	281	1727	133	613	9.3%	0.70 [0.56, 0.88]
Rodriguez, Ecuador-2015	231	2510	421	4295	9.8%	0.93 [0.79, 1.10]
Bedolla-Barajas, Mexico-2017	77	814	19	189	6.6%	0.93 [0.55, 1.59]
Solé, Brazil_Pernambuco-2007	512	3066	468	3057	9.9%	1.11 [0.97, 1.27]
Cooper, Ecuador-2016	394	1458	141	611	9.4%	1.23 [0.99, 1.54]
Menezes, Brazil-2015	2301	50015	316	10187	10.0%	1.51 [1.34, 1.70]
Soares, Brazil-2004	541	3340	36	330	8.2%	1.58 [1.10, 2.26]
Solé, Brazil_Rio Grande-2007	497	2674	44	352	8.4%	1.60 [1.15, 2.22]
Han, Argentina-2017	198	1525	12	168	5.9%	1.94 [1.06, 3.56]
Kausel, Chile-2013	512	3264	6	100	4.3%	2.91 [1.27, 6.69]
Gaviola, Peru-2016	99	1501	30	1452	7.6%	3.35 [2.21, 5.07]
Lynch, Venezuela-1984	25	431	6	336	3.9%	3.39 [1.37, 8.35]
Robinson, Peru-2011	73	725	20	716	6.8%	3.90 [2.35, 6.46]
**Total (95% CI)**		**73050**		**22406**	**100.0%**	**1.52 [1.22, 1.90]**
Total events	5741		1652			

Heterogeneity: Tau² = 0.13; Chi² = 105.11, df = 12 (P < 0.00001); I² = 89%
Test for overall effect: Z = 3.67 (P = 0.0002)
