# Supplementary material for: Urbanisation and asthma in low-income and middle-income countries: a systematic review of the urban–rural differences in asthma prevalence
Source: Thorax. 2019 Jul 5;74(11):1020–30. doi: 10.1136/thoraxjnl-2018-211793 (PMC6860411; doi:10.1136/thoraxjnl-2018-211793)
Supplement: Supplementary data [file thoraxjnl-2018-211793supp002.pdf]

## Supplementary Tables

### Supplementary Table 1. Re-classification of publications using more than one category to define urban or rural settings in asthma studies.

| Publication | Asthma definition (AD) | Categories | New Categories | AD 1 (%) | AD 2 (%) | AD 3 (%) | Urban vs rural Prevalence (%) |
|---|---|---|---|---|---|---|---|
| Addo-Yobo et al. (10) Ghana, 2007 | AD1. Exercise challenge test | Urban Rich | Urban | 8.3 | | | 5.65 vs 3.9 |
| | | Urban Poor | Urban | 3 | | | |
| | | Rural | Rural | 3.9 | | | |
| Addo-Yobo et al. (28) Ghana, 1997 | AD1. Exercise challenge test | Urban Rich | Urban | 4.2 | | | 3.42 vs 2.2 |
| | | Urban Poor | Urban | 1.4 | | | |
| | | Rural | Rural | 2.2 | | | |
| Bouayad et al. (59) Morroco, 2006 | AD1. Current wheeze | Casablanca city | Urban | 16.2 | 23.9 | | 9.91 vs 6.44 |
| | AD2. Wheezing ever | Marrakech city | Urban | 4.6 | 10.3 | | 16.13 vs 12.9 |
| | | Ben Slimane city | Urban | 8.9 | 14.2 | | |
| | | Boulmane rural area | Rural | 6.4 | 12.9 | | |
| El-Sharif et al. (33) Palestine, 2002 | AD1. Current wheeze | Refugee camps | Urban | 12.6 | 22.1 | 15.6 | 9.9 vs 7.46 |
| | AD2. Wheeze ever | Cities | Urban | 7.2 | 16.5 | 7.3 | 19.3 vs 16.6 |
| | AD3. Doctor diagnosis | Semi urban | Rural | 6.3 | 12.9 | 5.6 | 11.45 vs 8.5 |
| | | Typical rural | Rural | 10.2 | 18.1 | 10 | |
| | | Primitive | Rural | 5.9 | 15.8 | 9.9 | |
| Keeley rt al. (32) Zimbabwe, 1991 | AD1. Exercise challenge test | Urban North | Urban | 5.8 | | | 4.45 vs 0.15 |
| | | Urban South | Urban | 3.1 | | | |
| | | Rural | Rural | 0.01 | | | |
| Steiman et al. (31) South Africa, 2003 | AD1. Exercise challenge test | Urban School | Urban | 32.9 | | | 33.65 vs 17 |
| | | Recently urbanized | Urban | 34.4 | | | |
| | | Rural school | Rural | 17 | | | |
| Viinamen et al. (24) Mongolia, 2005 | AD1. Questionnaire diagnosis | City | Urban | 2.1 | | | 2.1 vs 1.75 |
| | | Rural towns | Rural | 2.4 | | | |
| | | Villages | Rural | 1.1 | | | |
| Gaur et al. (29) India, 2006 | AD1. Exercise challenge test | Urban city | Urban | 7.7 | | | 10.21 vs 13. 3 |
| | | Urban slum | Urban | 11.9 | | | |
| | | Rural | Rural | 13.3 | | | |
| Mavale-Manuel et al (27) Mozambique, 2007 | AD1. Current wheeze | Urban | Urban | 13.6 | 25.15 | | 13.5 vs 12.8 |
| | AD2. Wheeze ever | Suburban | Urban | 13.5 | 24 | | 24.58 vs 22.6 |
| | | Semirural | Rural | 12.9 | 22.6 | | |
| Kausel et al.(26) Chile, 2013 | AD1. Current wheeze | Urban | Urban | 16 | 15.8 | | 15.69 vs 6 |
| | AD2. Self-reported asthma | Semiurban | Urban | 10 | 17.6 | | 16 vs 20 |
| | | Rural | Rural | 6 | 20 | | |
| Gaviola et al.(24) Peru, 2016 | AD1. Current wheeze | Urban_Lima | Urban | 9.9 | 8.2 | | 5.85 vs 1.85 |
| | AD2. Doctor diagnosis | Urban_Puno | Urban | 1.8 | 2.6 | | 5.4 vs1.15 |
| | | Semiurban_Tumbes | Rural | 2.5 | 1.7 | | |
| | | Rural_Puno | Rural | 1.2 | 0.6 | | |

**Supplementary Table 2. Differences in asthma prevalence between urban and rural populations by asthma definition.**

| | | Wheeze ever | | Current Wheeze | | Doctor diagnosis | | Exercise test | | Self-reported | | Questionnaire | |
|---|---|---|---|---|---|---|---|---|---|---|---|---|---|
| **Author (reference)** | **Country, year** | **Urban vs Rural** | **diff** | **Urban vs Rural** | **diff** | **Urban vs Rural** | **diff** | **Urban vs Rural** | **diff** | **Urban vs Rural** | **diff** | **Urban vs Rural** | **diff** |
| Addo-Yobo et al.(10) | Ghana, 2007 | | | | | | | 5.7 vs 3.9 | ** | | | | |
| Selcut et al. (11) | Turkey, 2010 | | | 6 vs 5.2 | ** | | | | | | | 5.8 vs 5.2 | ** |
| Selcut et al. (11) | Turkey, 2010 | | | 12.6 vs 9.7 | *** | | | | | | | 12.1 vs 8.6 | *** |
| Oluwole et al.(57) | Nigeria , 2013 | | | | | | | | | | | 8 vs 7.5 | ** |
| Yang et al. (73) | China , 2015 | | | | | | | | | | | 6.6 vs 2.5 | *** |
| Kahwa et al. (77 ) | Jamaica, 2012 | | | 18.5 vs 20.5 | * | 18.3 vs 15.4 | ** | | | | | | |
| Mavale-Manuel et al. (27) | Mozambique. 2007 | 24.6 vs 22.4 | ** | 13.5 vs 12.8 | ** | | | | | | | | |
| Hijazi et al. (67) | Saudi Arabia, 1998 | 17.8 vs 7.3 | *** | 13.2 vs 6.4 | *** | | | | | 14.9 vs 5.4 | *** | | |
| Walraven et al. (54) | Gambia, 2001 | | | 4.2 vs 3.3 | ** | 3.6 vs 0.7 | *** | | | | | | |
| Solis et al. (78) | Bolivia, 2014 | 27.4 vs 34.9 | * | 16.4 vs 21.7 | * | | | 19.2 vs 13.1 | ** | | | | |
| Solé et al. (22) | Brazil, 2007 | 32.6 vs 23 | *** | 18.6 vs 12.5 | *** | | | | | 21.1 vs 8.5 | *** | | |
| Solé et al. (22) | Brazil, 2007 | 42.1 vs 37.4 | *** | 16.7 vs 15.3 | ** | | | | | 14.9 vs 11.1 | *** | | |
| Steiman et al. (31) | South Africa, 2003 | | | | | | | 33.7 vs 17 | *** | | | | |
| Kausel et al. (26) | Chile, 2013 | | | 15.7 vs 6 | *** | | | | | 16 vs 20 | * | | |
| Zhang et al. (68) | China, 2014 | | | | | | | | | | | 1.1 vs 0.5 | *** |
| Keeley et al. (32) | Zimbabwe, 1991 | | | | | | | 4.5 vs 0.2 | *** | | | | |
| Robinson et al. (79) | Peru, 2011 | | | 10.1 vs 2.8 | *** | 13 vs 2.2 | *** | | | 11.6 vs 3.1 | *** | | |
| Abdalla et al. (55) | Egypt, 2012 | | | | | 6.4 vs 5.2 | ** | | | | | | |
| Yemaneberhan et al. (56) | Ethiopia, 1997 | | | 3.7 vs 1.2 | *** | | | | | 3.6 vs 1.3 | *** | | |
| Addo-Yobo et al . (28) | Ghana,1997 | | | | | | | 3.4 vs 2.2 | ** | | | | |
| Vlaski et al. (85) | Macedonia, 2014 | | | 7.2 vs 4.9 | *** | | | | | 1.9 vs 1.2 | ** | | |
| El-Sharif et al. (33) | Palestine, 2002 | 19.3 vs 15.6 | *** | 9.9 vs 7.5 | *** | 11.5 vs 8.5 | *** | | | | | | |
| Adetoun et al.(43) | Nigeria,2013 | | | 5.7 vs 4.1 | ** | 0.4 vs 2.9 | * | | | | | | |
| Viinanen et al. (30) | Mongolia, 2005 | | | | | | | | | | | 2.1 vs 1.8 | ** |
| Gaur et al.(29) | India, 2006 | | | | | | | 10.2 vs 13.3 | * | | | | |
| Ng'ang'a et al. (44) | Kenya, 1998 | | | | | | | 22.9 vs 13.2 | *** | | | | |
| Musafiri et al. (45) | Rwanda, 2011 | | | 11.9 vs 13.1 | * | 7.3 vs 5.7 | ** | | | | | 19.3 vs 8.3 | *** |
| Calvert et al. ( 46) | South Africa, 2015 | | | | | | | 14.9 vs 8.7 | *** | | | | |
| Shimwela et al. (47) | Tanzania, 2014 | | | 23.1 vs 12.1 | *** | | | 6.3 vs 2.4 | *** | 17.1 vs 6.6 | *** | | |
| Guner et al. (69) | Turkey, 2011 | | | 5.6 vs 6.3 | * | 10.5 vs 7.1 | ** | | | | | 12.7 vs 9.9 | ** |
| Fedortsiv et al. (70) | Ukraine, 2012 | | | 12.4 vs 10.6 | ** | 2.6 vs 1.3 | *** | | | | | | |
| Lâm et al. (71) | Vietnam, 2011 | 5.6 vs 3.9 | *** | 4.3 vs 5.1 | * | 3.9 vs 3.8 | ** | | | | | | |

| | | | | | | | | | | | | | |
|---|---|---|---|---|---|---|---|---|---|---|---|---|---|
| Dagoye et al. (48) | Ethiopia, 2003 | | | 4.4 vs 2 | *** | | | | | | | | |
| Ekici et al. (72) | Turkey 2009 | | | 6.9 vs 9.9 | * | | | | | | | 11.4 vs 14.1 | * |
| Rodriguez et al. (80) | Ecuador, 2015 | | | 9.4 vs 10.1 | * | | | | | | | | |
| Van Niekerk et al.A (49) | South Africa, 1979 | | | | | | | 3.2 vs 0.1 | *** | | | | |
| Hasan et al. (58) | Palestine, 2000 | 16.4 vs 12 | ** | 10.5 vs 5.5 | *** | | | | | 4.2 vs 2.8 | ** | | |
| Odhiambo et al. (50) | Kenya, 1998 | 13.4 vs 3.2 | *** | 9.5 vs 2.5 | *** | 4.2 vs 0.2 | *** | | | | | | |
| Feng et al. (59) | China, 2016 | 14.6 vs 4.2 | *** | 6.1 vs 1.5 | *** | 6.9 vs 3.4 | *** | | | | | | |
| Paranesh et al. (60) | India, 2002 | | | | | | | | | | | 16.6 vs 5.7 | *** |
| Soares et al. (81) | Brazil, 2004 | 37 vs 23.6 | *** | 16.2 vs 10.9 | *** | | | | | 24.7 vs 15.2 | *** | | |
| Ma Y et al. (61) | China, 2009 | | | 7.2 vs 1 | *** | 6.3 vs 1.1 | *** | | | | | | |
| Huang et al. (62) | China, 2014 | 6.6 vs 3.2 | *** | 2.8 vs 1.5 | *** | | | | | | | | |
| Cooper et al. (82) | Ecuador, 2016 | 27 vs 23 | ** | | | | | | | | | | |
| Bedolla et al. (83) | Mexico, 2017 | 23.5 vs 24.9 | * | 9.5 vs 10.1 | * | 8.5 vs 5.8 | ** | | | | | | |
| Zhu et al. (63) | China, 2015 | | | | | | | | | | | 3.7 vs 1.3 | *** |
| Chakravarthy et al. (64) | India, 2002 | 19.6 vs 14.4 | ** | | | 5.4 vs 4.5 | ** | | | | | | |
| Menezes et al. (74) | Brasil, 2015 | | | | | 4.6 vs 3.1 | *** | | | | | | |
| Tug et al. (65) | Turkey, 2002 | 25.8 vs 29.6 | * | 19.3 vs 27.3 | * | | | | | | | | |
| Zedan et al. (51) | Egipt, 2009 | | | | | | | | | | | 8 vs 7 | ** |
| Mugusi et al. (23) | Cameroon, 2004 | 5.6 vs 2.9 | *** | 3.4 vs 2.9 | ** | | | | | 3.7 vs 3.2 | ** | | |
| Mugusi et al. (23) | Cameroon, 2004 | 2.3 vs 3.7 | * | 1 vs 3.3 | * | | | | | 1.2 vs 3.3 | * | | |
| Gaviola et al. (24) | Peru, 2016 | | | 11 vs 3.1 | *** | 6.3 vs 1.3 | *** | | | | | | |
| Yakubovich et al.E (52) | South Africa, 2016 | | | | | | | | | 5.9 vs 4 | *** | | |
| Kumar et al. (66) | India, 2017 | | | | | | | | | | | 11.9 vs 9.8 | ** |
| Han et al. (75) | Argentina, 2017 | | | 13 vs 7.1 | *** | 4.3 vs 4.8 | * | | | | | | |
| Ehrlich et al. (53) | South Africa, 2005 | | | 15.3vs17.9 | * | 4.2vs3.1 | *** | | | | | | |
| Lynch et al. (76) | Venezuela, 1984 | | | | | 5.8 vs 1.8 | *** | | | | | | |
| Bouayad et al. (25) | Morroco, 2006 | 16.13 vs12.9 | *** | 9.9 vs 6,4 | *** | | | | | | | | |

*****Higher in urban area and statistical significant. ** Higher in urban area and no statistical significant, * Higher in rural area.**

**Supplementary Table 3. Methodological quality of studies included in systematic review**

| Author (Country-year of publications) | Area description | Population description | Sample method | Sample size | Urban definition | Response rate | Overall quality |
|---|---|---|---|---|---|---|---|
| Van Niekerk et al. (South Africa-1979) | + | + | - | + | + | unk | Low |
| Lynch et al. (Venezuela-1984) | + | + | - | - | + | unk | Low |
| Keeley et al. (Zimbabwe-1991) | + | + | - | + | + | unk | Low |
| Addo-Yobo et al. (Ghana*-1997) | + | + | - | + | + | unk | Low |
| Yemaneberhan et al. (Ethiopia-1997) | - | - | + | + | + | unk | Low |
| Hijazi et al. (Saudi Arabia-1998) | + | - | + | + | + | unk | Low |
| Ng'ang'a et al. (Kenya-1998) | + | + | + | + | + | + | High |
| Odhiambo et al. (Kenya-1998) | + | + | + | + | + | + | High |
| Hasan et al. (Palestine-2000) | + | + | + | + | + | + | High |
| Walraven et al. (Gambia-2001) | + | + | + | + | + | + | High |
| Chakravarthy et al. (India-2002) | + | - | - | + | - | + | Low |
| El-Sharif et al. (Palestine-2002) | + | + | + | + | - | + | Medium |
| Paranesh et al. (India-2002) | + | + | - | + | - | unk | Low |
| Tug et al. (Turkey-2002) | + | + | - | + | + | + | Medium |
| Dagoye et al. (Ethiopia-2003) | - | - | - | + | + | unk | Low |
| Steiman et al. (South Africa-2003) | + | + | - | - | + | + | Low |
| Mugusi et al. (Tanzania-2004) | + | + | + | + | + | unk | Medium |
| Mugusi et al. (Cameroon-2004) | + | + | + | + | + | unk | Medium |
| Maia et al. (Brazil-2004) | + | + | + | + | + | unk | Medium |
| Ehrlich et al. (South Africa-2005) | - | + | + | + | + | + | Medium |
| Viinanen et al. (Mongolia-2005) | + | + | + | + | + | + | High |
| Bouayad et al. (Morroco-2005) | + | + | - | + | + | - | Low |
| Gaur et al. (India*-2006) | + | + | - | + | + | unk | Low |
| Addo-Yobo et al. (Ghana*-2007) | + | + | - | + | + | unk | Low |
| Cunha et al. (Brazil-2007) | - | + | + | + | + | na | Medium |
| Mavale-Manuel et al. (Mozambique-2007) | + | + | + | + | + | + | High |
| Solé et al. (Brazil^-2007) | + | + | + | + | + | unk | Medium |
| Solé et al. (Brazil^-2007) | + | + | + | + | + | unk | Medium |
| Ekici A et al. (Turkey-2009) | + | - | + | + | - | + | Low |
| Ma Y et al. (China-2009) | - | + | + | + | - | unk | Low |
| Zedan et al. (Egipt-2009) | - | - | + | + | + | + | Low |
| Calvert et al. (South Africa-2010) | + | + | + | + | + | unk | Medium |
| Selcuk et al. (Turkey*-2010) | + | + | + | + | + | + | High |
| Selcuk et al. (Turkey*-2010) | + | + | + | + | + | + | High |
| Guner et al. (Turkey-2011) | - | - | + | - | - | + | Low |
| Oluwole et al.(Nigeria-2013) | - | - | - | + | + | unk | Low |
| Yang et al. (China-2015) | - | - | - | + | + | unk | Low |
| Lam et al. (Vietnam-2011) | + | + | + | + | + | + | High |
| Musafiri et al. (Rwanda-2011) | + | + | + | + | + | + | High |
| Robinson et al. (Peru-2011) | + | + | - | + | + | + | Medium |
| Rodriguez et al. (Ecuador-2011) | + | + | + | + | + | na | High |
| Abdallah et al. (Egypt-2012) | + | + | + | + | + | + | High |
| Fedortsiv et al. (Ukraine-2012) | - | - | + | + | - | + | Low |
| Kahwa et al. (Jamaica-2012) | + | + | + | + | + | + | High |
| Adetoun Mustapha et al. (Nigeria-2013) | + | + | + | + | + | + | High |
| Kausel et al. (Chile-2013) | + | - | - | + | + | unk | Low |
| Antunes et al (Brazil-2014) | - | - | + | + | + | na | Low |
| Fatore et al. (Latin America-2014) | + | + | + | + | + | na | High |
| Huang et al. (China-2014) | + | + | - | + | + | unk | Low |
| Shimwela et al. (Tanzania-2014) | + | + | + | + | + | + | High |
| Solis et al. (Bolivia-2014) | + | + | + | + | + | + | High |
| Vlaski et al. (Macedonia-2014) | + | + | + | + | + | + | High |
| Zhang et al. (China-2014) | - | + | + | + | + | + | Medium |



Rodriguez A, *et al. Thorax* 2019; 0:1020–1030. doi: 10.1136/thoraxjnl-2018-211793

| | | | | | | | |
|---|---|---|---|---|---|---|---|
| Menezes et al. (Brasil-2015) | - | - | + | + | - | unk | Low |
| Rodriguez et al. (Ecuador-2015) | + | + | - | + | + | + | Medium |
| Cooper et al. (Ecuador-2016) | + | + | - | + | - | + | Low |
| Tabalipa et al. (Brazil-2015) | + | + | + | + | + | na | High |
| Feng, et al. (China-2015) | + | + | + | + | + | + | High |
| Zhu et al. (China-2015) | + | + | + | + | + | + | High |
| Yakubovich et al. (South Africa-2016) | + | + | + | + | + | unk | Medium |
| Dias et al. (Brazil-2016) | + | + | + | + | + | na | High |
| Ponte et al. (Brazil-2016) | - | + | + | + | + | na | Medium |
| Gaviola et al. (Peru-2016) | + | + | + | + | + | + | High |
| Bedolla-Barajas et al. (Mexico-2017) | + | + | + | + | + | + | High |
| Han et al. (Argentina-2017) | + | + | - | + | + | + | Medium |
| Kumar et al. (India-2017) | - | + | + | + | - | + | Low |

**(na) not applicable; (unk) unknown; (+) adequate; (-) not adequate**



Rodriguez A, *et al. Thorax* 2019; 0:1020–1030. doi: 10.1136/thoraxjnl-2018-211793
